# Activation of aldehyde dehydrogenase-2 improves ischemic random skin flap survival in rats

**DOI:** 10.3389/fimmu.2023.1127610

**Published:** 2023-06-27

**Authors:** Taotao Zhou, Xibin Wang, Kaitao Wang, Yi Lin, Zhefeng Meng, Qicheng Lan, Zhikai Jiang, Jianpeng Chen, Yuting Lin, Xuao Liu, Hang Lin, Shijie Wu, Dingsheng Lin

**Affiliations:** ^1^ Department of Hand and Plastic Surgery, The Second Affiliated Hospital and Yuying Children’s Hospital of Wenzhou Medical University, The Second School of Medicine, Wenzhou Medical University, Wenzhou, China; ^2^ Department of Pulmonary and Critical Care Medicine, The First Affiliated Hospital of Wenzhou Medical University, The First School of Clinical Medical, Wenzhou Medical University, Wenzhou, China

**Keywords:** ALDH2, Alda-1, random skin flaps, PINK1/Parkin-mediated mitophagy, ischemia/reperfusion injury, inflammation, angiogenesis

## Abstract

**Objective:**

Random skin flaps have many applications in plastic and reconstructive surgeries. However, distal flap necrosis restricts wider clinical utility. Mitophagy, a vital form of autophagy for damaged mitochondria, is excessively activated in flap ischemia/reperfusion (I/R) injury, thus inducing cell death. Aldehyde dehydrogenase-2 (ALDH2), an allosteric tetrameric enzyme, plays an important role in regulating mitophagy. We explored whether ALDH2 activated by N-(1,3-benzodioxol-5-ylmethyl)-2,6-dichlorobenzamide (Alda-1) could reduce the risk of ischemic random skin flap necrosis, and the possible mechanism of action.

**Methods:**

Modified McFarlane flap models were established in 36 male Sprague-Dawley rats assigned randomly to three groups: a low-dose Alda-1 group (10 mg/kg/day), a high-dose Alda-1 group (20 mg/kg/day) and a control group. The percentage surviving skin flap area, neutrophil density and microvessel density (MVD) were evaluated on day 7. Oxidative stress was quantitated by measuring the superoxide dismutase (SOD) and malondialdehyde (MDA) levels. Blood perfusion and skin flap angiogenesis were assessed *via* laser Doppler flow imaging and lead oxide-gelatin angiography, respectively. The expression levels of inflammatory cytokines (IL-1β, IL-6, and TNF-α), vascular endothelial growth factor (VEGF), ALDH2, PTEN-induced kinase 1 (PINK1), and E3 ubiquitin ligase (Parkin) were immunohistochemically detected. Indicators of mitophagy such as Beclin-1, p62, and microtubule-associated protein light chain 3 (LC3) were evaluated by immunofluorescence.

**Results:**

Alda-1 significantly enhanced the survival area of random skin flaps. The SOD activity increased and the MDA level decreased, suggesting that Alda-1 reduced oxidative stress. ALDH2 was upregulated, and mitophagy-related proteins (PINK1, Parkin, Beclin-1, p62, and LC3) were downregulated, indicating that ALDH2 inhibited mitophagy through the PINK1/Parkin signaling pathway. Treatment with Alda-1 reduced neutrophil infiltration and expressions of inflammatory cytokines. Alda-1 significantly upregulated VEGF expression, increased the MVD, promoted angiogenesis, and enhanced blood perfusion.

**Conclusion:**

ALDH2 activation can effectively enhance random skin flap viability *via* inhibiting PINK1/Parkin-dependent mitophagy. Moreover, enhancement of ALDH2 activity also exerts anti-inflammatory and angiogenic properties.

## 1 Introduction

Random skin flap transplantation is commonly used to repair tissue defects caused by trauma, burns, ulcers, and tumor resection (1, 2). Such flaps lack blood vessels, and can thus be easily rotated (or otherwise moved) to cover a defect. Unfortunately, all such flaps evidence some necrosis postoperatively (especially distal areas that may lack adequate blood perfusion). When perfusion falls by more than 50%, the flap may enter a dangerous “shock” phase (3). After blood flow is restored following a long period of ischemia, the inflammatory response and generation of reactive oxygen species (ROS) increase, triggering secondary damage. Insufficient blood perfusion, ischemia/reperfusion (I/R) injury, and inflammation are believed to play major roles in the aggravation of flap necrosis (4–6).

Aldehyde dehydrogenase-2 (ALDH2), a mitochondrial enzyme, detoxifies acetaldehyde generated from alcohol (7). ALDH2 alleviates I/R injury. Chen et al. (8) published important insights into the potential role played by ALDH2 in the treatment of myocardial I/R injury in rats. ALDH2 activation decreased myocardial infarction by 60% when injected into the left ventricle. N-(1,3-benzodioxol-5-ylmethyl)-2,6-dichlorobenzamide, (Alda-1) was shown to activate ALDH2, increasing the activity of the wild-type enzyme 2.1-fold and that of a mutant enzyme 11-fold. More recently, Alda-1 has been shown to effectively treat acute I/R injury of the liver, brain, and intestine (9–11). During I/R, accumulated ROS and ROS-induced aldehydes may provoke mitochondrial autophagy (mitophagy), associated with a multitude of cardiovascular disorders (12). Mitophagy is regulated by several mechanisms, including the PTEN-induced kinase 1 (PINK1)/E3 ubiquitin ligase (Parkin)-mediated pathway (13). Alda-1 exerts cardioprotective effects by suppressing excessive activation of PINK1/Parkin-mediated mitophagy (14). In addition, Alda-1 lowers ROS levels, inflammasome expression, and the inflammatory response of alveolar epithelial cells (15). Alda-1 prevented vascular damage caused by amyloid β peptides in Alzheimer’s disease by reducing endothelial cell apoptosis, maintaining cell migration, and restoring angiogenesis (16).

Given the potential anti-I/R injury, anti-mitophagy, anti-inflammatory, and angiogenic properties of the Alda-1-mediated enhancement of ALDH2 activity, Alda-1 might well-treat random flap necrosis. We explored this possibility, and the mechanism in play.

## 2 Materials and methods

### 2.1 Ethics statement

All experimental procedures were in strict compliance with the Guide for the Care and Use of Laboratory Animals and the “3R” principles (replacement, reduction, and refinement). Ethical approval was obtained from the Ethics Committee for Experimental Animals of Wenzhou Medical University (approval no. WYDW 2022-0511). All rats were kept in separate cages at a comfortable temperature (21–27°C), appropriate humidity (40–60%), and with good ventilation and a regular circadian rhythm. General anesthesia (40 mg/kg of 1% [w/v] sodium pentobarbital delivered intraperitoneally [i.p.]) ensured that the rats felt no pain during surgery. All rats were euthanized using a pentobarbital overdose 7 days after surgery. Thus, the number of rats used and the suffering of the animals were minimized.

### 2.2 Reagents and animals

Alda-1 (purity ≥ 98%) was purchased from Good Laboratory Practice Bioscience (Montclair, IL, USA). Superoxide dismutase (SOD) activity and the malondialdehyde (MDA) content were measured using commercial assay kits (Jiangsu Jiancheng Technology Co., Ltd., Nanjing, China). The following antibodies were purchased from Affinity Biosciences (Cincinnati, OH, USA): anti-interleukin (IL)-1β (Cat# AF5103), anti-IL-6 (Cat# DF6087), anti-tumor necrosis factor (TNF)-α (Cat# AF7014), anti-vascular endothelial growth factor (VEGF) (Cat# AF5131), anti-ALDH2 (Cat# DF6358), anti-PINK1 (Cat# DF7742), anti-Parkin (Cat# AF0235), anti-Beclin-1(Cat#AF5128), anti-p62(Cat#AF5384), and anti-LC3(Cat#AF4650). Immunohistochemistry kits were purchased from Maixin Biotechnology Development Co. Ltd. (Fuzhou, China). Pathogen-free male Sprague-Dawley rats (n = 36, 200–250 g each, and 2–3 months of age) were supplied by Zhejiang Vital River Laboratory Animal Technology Co. Ltd. (Jiaxing, China).

### 2.3 Surgical procedures and drug administration

**Figure 1:**
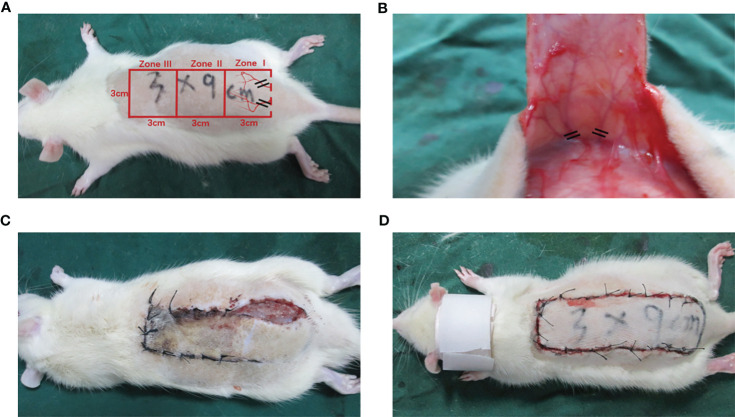
The establishment of modified McFarlane skin flap model on the back of each rat. **(A)** Diagram of designed incision and subcutaneous illiac ateries **(B)** Exposure and disconnection of iliac blood vessels **(C)** Self-biting behavior was identified by pre-experiments **(D)** Application of cervical collars after operation.

The 36 rats were randomly assigned to three different groups: the low-dose Alda-1 group, high-dose Alda-1 group, and control group. Each rat was injected i.p. with 1% (w/v) sodium pentobarbital (40 mg/kg) before surgery and placed in the prone position. A modified McFarlane, ischemic random skin flap was placed in all rats (17) (**Figure 1A**). In brief, after removing the back hair, the skin was cut to yield a long rectangular flap (9 × 3 cm). The long cut ran parallel to the spine and the short cut parallel to the iliac crest; the skin and subcutis were carefully separated from the deep fascia. The two symmetrical iliac arteries were ligated, the subdermal capillary network was conserved, and the bleeding was completely halted (**Figure 1B**). Finally, the detached flap was sutured (primarily with 4.0 nylon) and the wound treated with iodine. All flaps were divided into three zones (I, II, and III) along the proximal–distal axis.

After surgery, a protective device that resembled a cervical vertebra tractor was placed in each rat to prevent flap biting; all rats were kept in separate cages (**Figures 1C, D**). In line with the manufacturer’s instructions, Alda-1 was dissolved in 10% dimethyl sulfoxide (DMSO) and 90% corn oil (both v/v) to 2.5 mg/mL. The doses, route and frequency of drug administration were selected according to the preliminary experiment and other previous studies (9–11). The experimental animals intraperitoneally received Alda-1 for 7 successive days at 10 mg/kg/day in the low-dose group and 20 mg/kg/day in the high-dose group. The control group was treated with 10% DMSO and 90% corn oil (the same amounts, route and frequency). All rats were euthanized on day 7 and tissue samples collected.

### 2.4 General observations and flap viability measurements

We recorded the flap color, elasticity, hair growth, and tissue necrosis. All flaps were photographed. Image-Pro Plus ver. 6.0 (Media Cybernetics, Rockville, MD, USA) was used to outline the survival areas. The percentage survival was: (survival area/total area) × 100%.

### 2.5 Histopathological evaluation

On day 7, rats were euthanized with sodium pentobarbital. Tissue samples (n = 6, 1 × 1 cm) were obtained from zone II of each flap, followed by immersion in paraformaldehyde for 24 h, coating with paraffin, and slicing (4-μm-thick sections). Inflammatory cell infiltration, tissue edema, and cell damage were evaluated under a light microscope (VHX-7000 Milton Keynes, Keyence, UK) at ×100 magnification. The number of neutrophils and microvessel cross-sections per unit area (/mm^2^) were calculated at ×200 magnification; we recorded the level of neutrophil infiltration and the microvascular density (MVD).

### 2.6 Lead oxide-gelatin angiography

Each rat was placed supine and a 22-gauge indwelling needle was inserted into the common carotid artery (on one side) to establish arterial access. The blood was completely drained, and a gelatin-lead oxide mixture (100 mL/kg) was injected until the ends of the extremities acquired the color of the solution. The specimens were held at –20°C for 12 h, after which X-ray imaging (Bucky Diagnost CS, Philips Medical Systems DMC GmbH, Germany) was used to observe dorsal flap angiogenesis.

### 2.7 Laser Doppler imaging

Blood flow in zones I–III was measured using a laser Doppler blood flowmeter (LDF) (Moor Instruments, Axminster, UK) before the rats were euthanized on day 7. We derived the perfusion volume (PU), thus the microcirculatory blood flow. Data were continuously sampled, and Moore LDI Review ver. 6.1 software (Moor Instruments, Axminster, UK) was used for data analysis. The PUs distinguished necrotic and surviving flap areas.

### 2.8 SOD activity and the MDA content

Zone II tissue samples were assayed in terms of SOD and MDA levels (measures of oxidative stress). SOD scavenges superoxide radicals (18) and MDA is a marker of lipid peroxidation. The SOD activity and MDA content were determined using the xanthine oxidase (19) and thiobarbituric acid (TBA) methods (20), respectively.

### 2.9 Immunohistochemical staining

Flap samples were deparaffinized in one-step rapid dewaxing solution and heat-induced antigen retrieval was performed for 3 min at 210°C. After cooling to room temperature, the specimens were held in endogenous peroxidase blocking solution for 20 min, incubated with primary antibodies overnight at 4°C [anti-IL-1β (1:150), -IL-6 (1:150), -TNF-α (1:150), -VEGF (1:150), -ALDH_2_ (1:150), -PINK1 (1:100), and -Parkin (1:100)], incubated with secondary antibodies, stained with a diaminobenzidine (DAB) solution, counterstained with hematoxylin, and imaged at 200× magnification under a light microscope. The photographs were analyzed using ImageJ software (National Institutes of Health, Bethesda, MD, USA).

### 2.10 Immunofluorescence

After deparaffinized at 60 °C for 12 h and heated with citric acid buffer for 3 min at 210°C, the sections were incubated with anti-Beclin-1(1:50), anti-p62 (1:100), and anti-LC3 (1:100) at 4°C overnight. The sections were then incubated with fluorescent secondary antibody. After the nuclei were stained with 4′, 6-diamidino-2- phenylindole (DAPI), slides were observed under a confocal microscope (VS200, Olympus Optical Co., Ltd., Tokyo, Japan).

### 2.11 Statistical analyses

All statistical analyses were performed using IBM SPSS ver. 26.0 software (IBM Corp., Armonk, NY, USA). Each datum is a mean ± standard error of the mean (SEM). The means of the three groups were compared using a one-way analysis of variance (ANOVA). A p-value < 0.05 indicated statistical significance.

## 3 Results

### 3.1 Alda-1 increased flap survival

**Figure 2:**
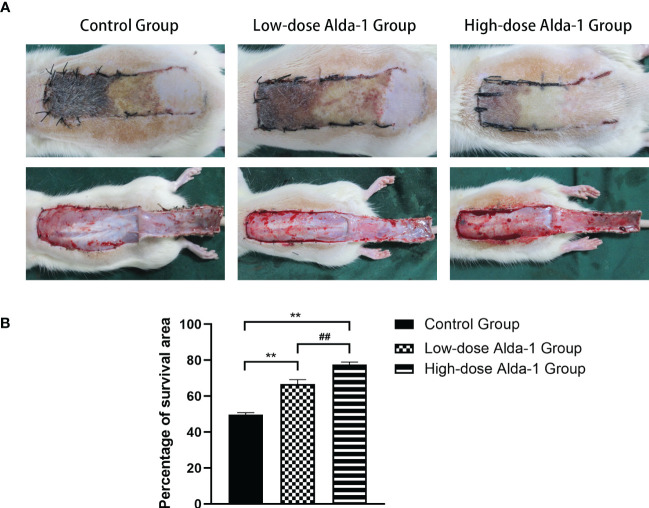
**(A)** Digital photographs recorded the condition of skin flaps on postoperative day 7(both outer surface and inner surface). **(B)** Comparison of survival area percentages among three groups (n=6). Data are represented as mean ± SEM. **P<0.01, vs. control group; ^##^P<0.01, low-dose Alda-1 group vs. high-dose Alda-1 group.

On day 7 after surgery, necrosis in the proximal region of the flap (zone I) was insignificant, zone II was partially necrotic, and the distal region (zone III) significantly necrotic. The boundaries between necrotic and surviving areas were macroscopically demarcated. Necrotic tissue was shrunken, stiff, and dry; brownish-black in color; and no hair grew. Surviving flap regions were fresh, soft, and ruddy, with tiny hairs. All flaps were then detached along the lines of the original incisions. **Figure 2A** shows that the atrophied tissue exhibited transmural necrosis and no subcutaneous capillaries. In contrast, the capillaries formed dense and interlacing networks in the surviving areas.

The mean survival area percentages revealed that the flap survival rate in the control group (49.74 ± 1.08%) was significantly lower than in both the low-dose Alda-1 group (66.73 ± 2.47%) and the high-dose Alda-1 group (77.57 ± 1.42%) (both p < 0.01). Moreover, the difference between the low- and high-dose Alda-1 groups was significant (p < 0.01) (**Figure 2B**).

### 3.2 Alda-1 decreased neutrophil infiltration and increased the MVD

**Figure 3:**
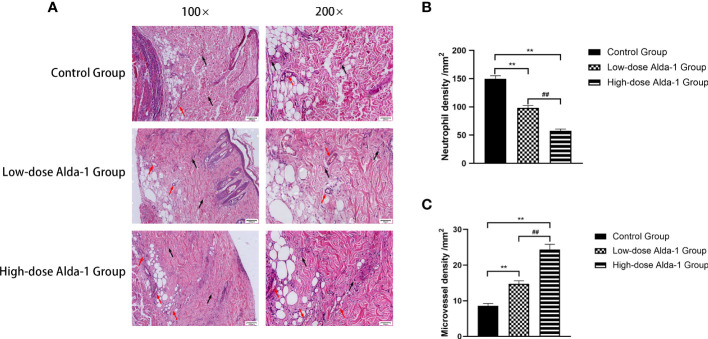
**(A)** Histopathological images of the H&E-stained pathological sections. All images were obtained at identical magnification, ×100 and ×200, scale bar = 100 μm and 50 μm. **(B)** Quantitative analysis of neutrophil infiltration among three groups (n = 6). Data are represented as mean ± SEM. **P<0.01, vs. control group; ^##^P<0.01, low-dose Alda-1 group vs. high-dose Alda-1 group. **(C)** Quantitative analysis of microvascular density among three groups (n = 6). Data are represented as mean ± SEM. **P<0.01, vs. control group; ^##^P<0.01, low-dose Alda-1 group vs. high-dose Alda-1 group.

The zone II regions of hematoxylin and eosin-stained tissue sections were analyzed under a light microscope; these regions contained both necrotic and surviving tissues (**Figure 3A**). Compared to the control group, the Alda-1 groups evidenced less disruption of tissue structure, less inflammatory cell exudation, and more neovascularization and fibroblast proliferation. Neutrophil infiltration in the high-dose Alda-1 group (57.54 ± 3.15/mm^2^) was significantly less than in the low-dose Alda-1 (98.25 ± 4.25/mm^2^, p < 0.01) and control (149.83 ± 5.38/mm^2^, p < 0.01) groups (**Figure 3B**). The MVD of the high-dose Alda-1 group was 24.39 ± 1.45/mm^2^, thus significantly higher than those of the low-dose Alda-1 (14.73 ± 0.86/mm^2^, p < 0.01) and control (8.60 ± 0.63/mm^2^, p < 0.01) groups (**Figure 3C**).

### 3.3 Alda-1 improved blood perfusion

**Figure 4:**
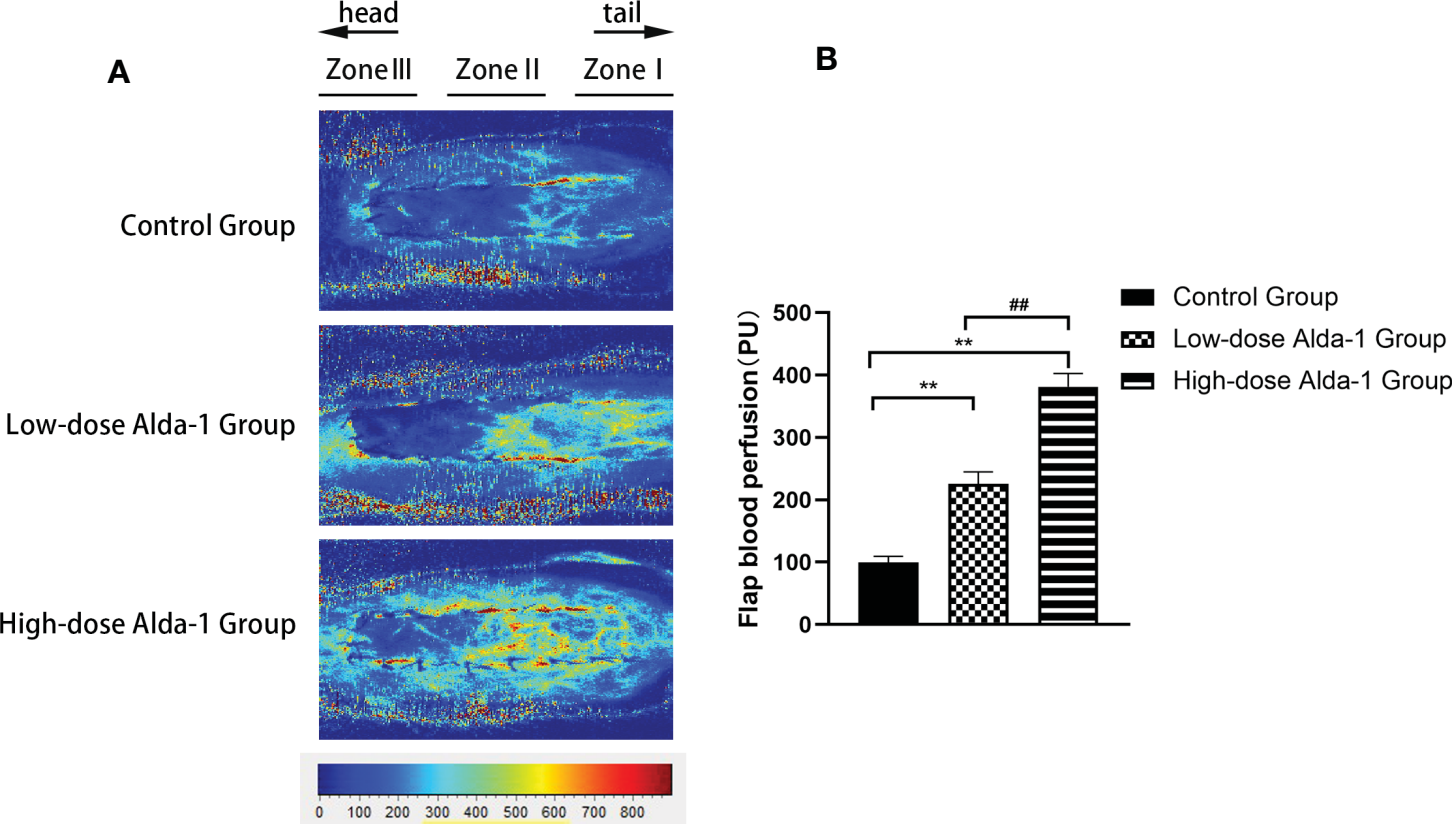
**(A)** Laser Doppler blood perfusion imaging showed blood flow in Zone I–III of flaps. **(B)** Comparison of flap blood perfusion among three groups (n=6). Data are represented as mean ± SEM. **P<0.01, vs. control group; ^##^P<0.01, low-dose Alda-1 group vs. high-dose Alda-1 group.

Laser Doppler perfusion imaging showed that the blood flow rates in the low- and high-dose Alda-1 groups (225.47 ± 19.19 and 381.62 ± 21.13 PU, respectively) were significantly higher than that in the control group (99.97 ± 9.32 PU; both p < 0.01) (**Figure 4**).

### 3.4 Alda-1 enhanced angiogenesis

**Figure 5:**
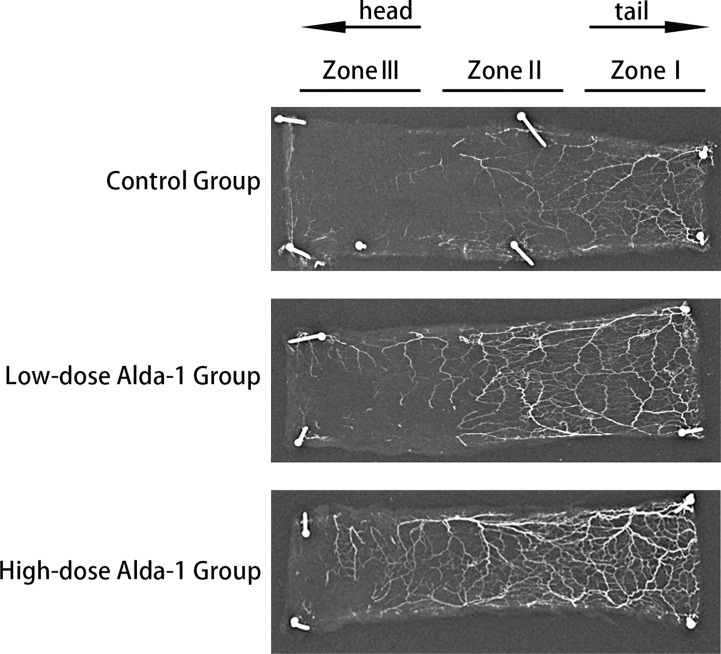
Images of X-ray angiography of skin flaps from three groups.

Lead oxide-gelatin angiography revealed that that the vascular density of the high-dose Alda-1 group was the greatest, followed by that of the low-dose Alda-1 group. The control group featured few blood vessels, particularly in the distal region (zone III) (**Figure 5**).

### 3.5 Alda-1 reduced oxidative stress

**Figure 6:**
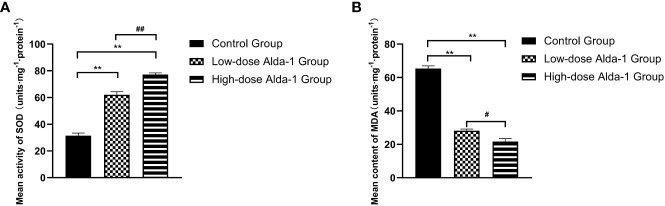
**(A)** Comparison of mean SOD activity among three groups on day 7 (n=6). Data are represented as mean ± SEM. **P<0.01, vs. control group; ^##^P<0.01, low-dose Alda-1 group vs. high-dose Alda-1 group. **(B)** Comparison of mean MDA content among three groups on day 7 (n=6). Data are represented as mean ± SEM. **P<0.01, vs. control group; ^#^P<0.05, low-dose Alda-1 group vs. high-dose Alda-1 group.

Seven days after surgery, the average SOD activity was considerably higher in the low-dose Alda-1 than in the control group (62.04 ± 2.34 and 31.44 ± 1.94 u·mg^-1^·protein^-1^, respectively, p < 0.01). The mean SOD activity of the high-dose Alda-1 group was 77.20 ± 1.23 u·mg^-1^·protein^-1^, thus significantly higher than that of the low-dose Alda-1 group (p < 0.01) (**Figure 6A**). The average MDA level was significantly lower in the low-dose Alda-1 group than in the control group (28.14 ± 1.01 and 65.38 ± 1.60 u·mg^-1^·protein^-1^, respectively, p < 0.01). The average MDA level of the high-dose Alda-1 group was 21.73 ± 1.70 u·mg^-1^·protein^-1^, thus significantly lower than that of the low-dose Alda-1 group (p < 0.05) (**Figure 6B**).

### 3.6 Alda-1 downregulated inflammatory cytokine levels

**Figure 7:**
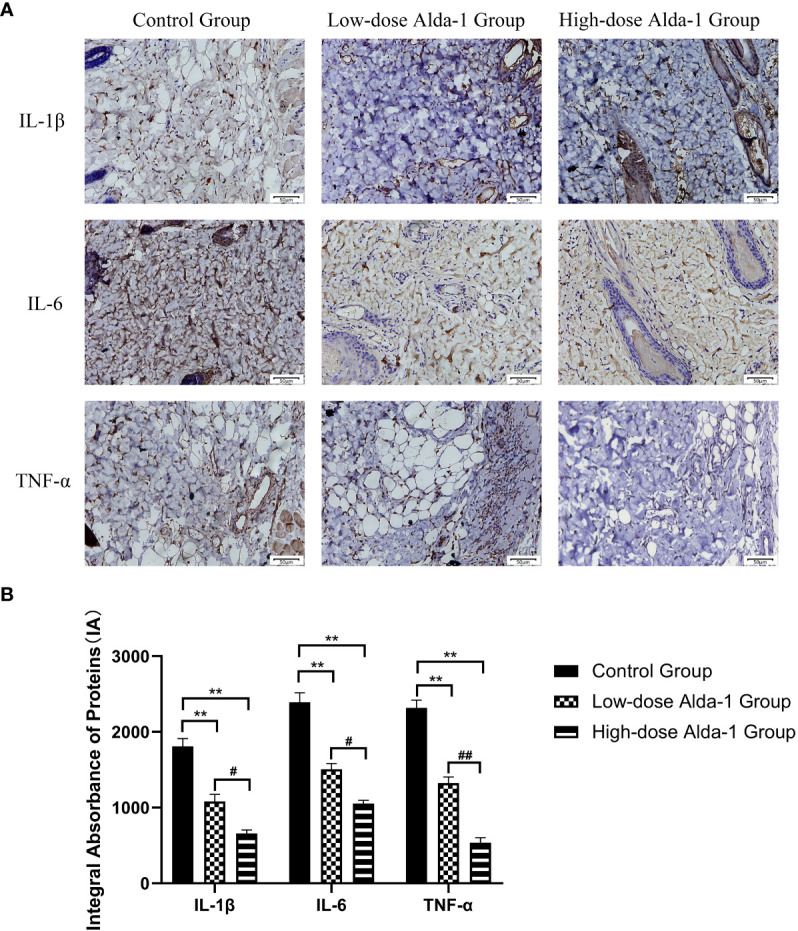
**(A)** Immunohistochemistry images of IL-1β, IL-6 and TNF-α. All images were obtained at identical mag-nification, ×200, scale bar = 50 μm. **(B)** Quantitative analysis of IL-1β, IL-6 and TNF-α content (n = 3). Data are represented as mean ± SEM. **P<0.01, vs. control group; ^#^P<0.05, low-dose Alda-1 group vs. high-dose Alda-1 group; ^##^P<0.01, low-dose Alda-1 group vs. high-dose Alda-1 group.

Immunohistochemical analyses revealed that the expression levels of IL-1β were significantly lower in the low- and high-dose Alda-1 groups (1,081.82 ± 92.78 IA and 658.48 ± 45.20 IA, respectively) than in the control group (1,808.33 ± 103.02 IA, both *p* < 0.01). The IL-6 expression levels were significantly lower in the low- and high-dose Alda-1 groups (1,505.67 ± 75.20 IA and 1,053.84 ± 42.62 IA, respectively) than in the control group (2,390.18 ± 126.42 IA, *p* < 0.01). The TNF-α expression levels were significantly lower in the low- and high-dose Alda-1 groups (1,323.78 ± 79.83 IA and 535.84 ± 64.66 IA, respectively) than in the control group (2,316.09 ± 102.00 IA, p< 0.01) (**Figure 7**).

### 3.7 Alda-1 upregulated VEGF expression

**Figure 8:**
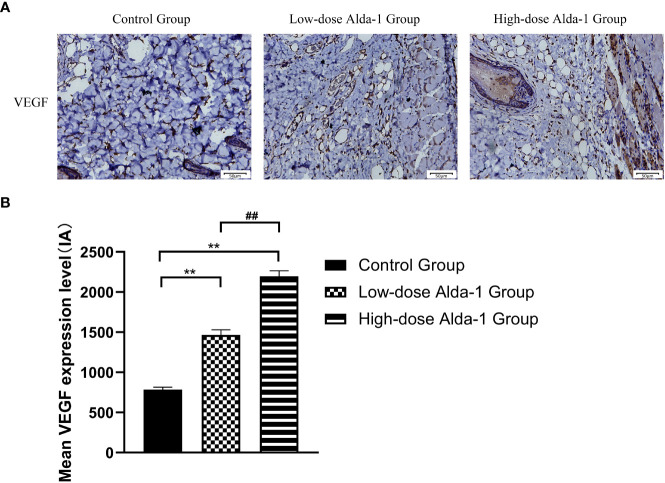
**(A)** Immunohistochemistry images of VEGF. All images were obtained at identical magnification, ×200, scale bar = 50 μm. **(B)** Quantitative analysis of VEGF content (n = 3). Data are represented as mean ± SEM. **P<0.01, vs. control group; ^##^P<0.01, low-dose Alda-1 group vs. high-dose Alda-1 group.

Immunohistochemical staining showed that, compared to the control group (784.30 ± 31.09 IA), the expression levels of VEGF in the low- and high-dose Alda-1 groups (1,466.16 ± 64.24 and 2,196.67 ± 68.59 IA, respectively, *p* < 0.01) were substantially higher (**Figure 8**).

### 3.8 Alda-1 inhibited the PINK1/Parkin signaling pathway

**Figure 9:**
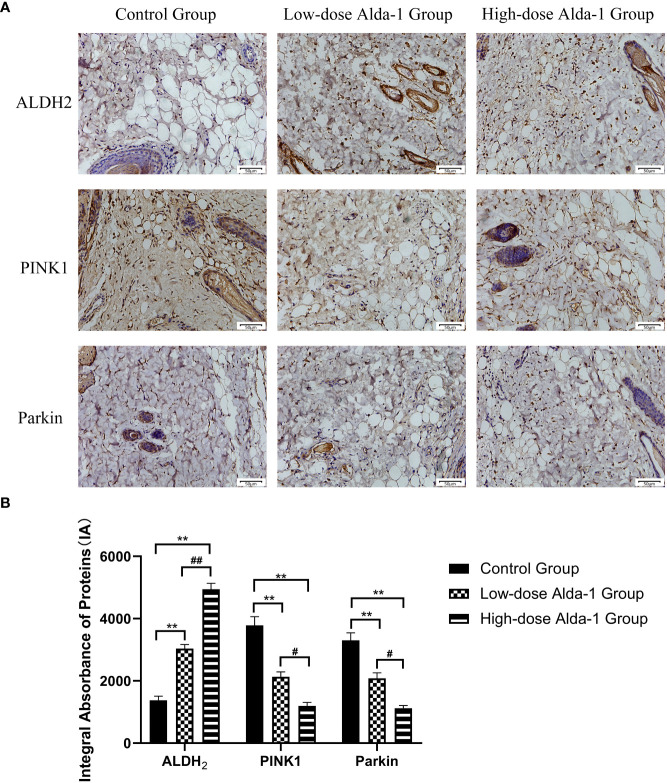
**(A)** Immunohistochemistry images of ALDH2, PINK1 and Parkin. All images were obtained at identical magnification, ×200, scale bar = 50 μm. **(B)** Quantitative analysis of ALDH2, PINK1 and Parkin content (n = 3). Data are represented as mean ± SEM. **P<0.01, vs. control group; ^##^P<0.01, low-dose Alda-1 group vs. high-dose Alda-1 group; ^#^P<0.05, low-dose Alda-1 group vs. high-dose Alda-1 group.

Immunohistochemical staining (**Figure 9A**) showed that, in the Alda-1 groups, ALDH2 expression markedly increased whereas the levels of key mitophagy factors (including PINK1 and Parkin) were greatly reduced. As shown in **Figure 9B**, the ALDH2 expression levels were significantly higher in the low- and high-dose Alda-1 groups (3,036.71 ± 131.28 IA and 4,943.19 ± 189.14 IA, respectively) than in the control group (1,378.11 ± 130.42 IA, p < 0.01). The PINK1 levels were significantly lower in the low- and high-dose Alda-1 groups (2,131.44 ± 158.04 IA and 1,196.73 ± 112.08 IA, respectively) than in the control group (3,783.59 ± 280.47 IA, p < 0.01). The Parkin levels were significantly lower in the low- and high-dose Alda-1 groups (2,082.48 ± 173.85 IA and 1,121.49 ± 84.62 IA, respectively) than in the control group (3,301.56 ± 240.78 IA, p < 0.01). For all protein biomarkers, significant differences between the low- and high-dose Alda-1 groups were also apparent (all p < 0.05).

### 3.9 Inhibition of PINK1/Parkin signaling pathway decreased mitophagy-related proteins

**Figure 10:**
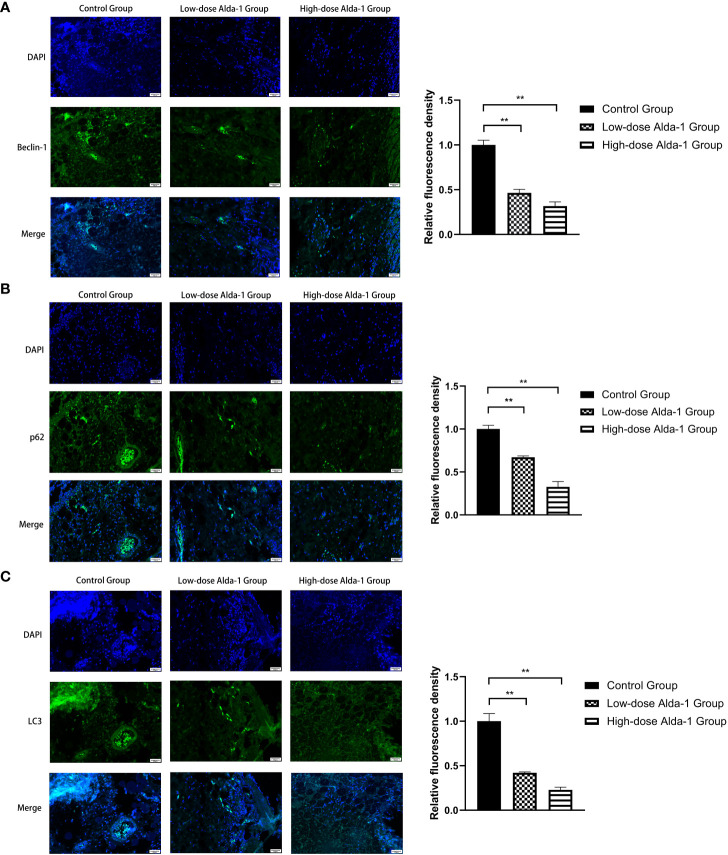
Immunofluorescence images of Beclin-1 **(A)**, p62 **(B)**, and LC3**(C)** were captured under fluorescence microscopy, and the relative fluorescence intensity was calculated. All images were obtained at identical magnification, ×200, scale bar = 50 μm. Data are represented as mean ± SEM (n = 3). **P<0.01, vs. control group.

As shown in **Figure 10**, we found that the fluorescence intensity of biomarkers of mitophagy (Beclin-1, p62, and LC3) in the low- and high-dose Alda-1 groups was lower than that in the control group. The result indicates that the mitophagy activity was weakened remarkably after treatment with Alda-1.

## 4 Discussion

The most common (and most serious) complication after flap transplantation is distal necrosis of the flap. In such a case, a second operation may be required, increasing the hospital stay, the financial burden on the patient, and psychological stress (on both the surgeon and the patient); these factors limit the clinical utility of flap transplantation. For decades, efforts have been made to understand the (complex) pathophysiological mechanism of flap necrosis. Many studies have confirmed that I/R injury and inflammation are risk factors and VEGF-mediated angiogenesis is protective. I/R (a common pathological process) is believed to be primarily responsible for the necrosis of ischemic skin flaps (21). ROS, by-products of oxidative phosphorylation, are produced (principally) by the electron transport chain of mitochondria (22). Normally, ROS production and removal are in dynamic balance; this maintains cellular homeostasis (23). When the blood supply is insufficient, and blood reperfusion then commences, the electron transport chain is disrupted and the mitochondria damaged, triggering an ROS burst. Excessive ROS damage macromolecules, including DNA, proteins, and lipids; DNA is mutated, proteins oxidized, lipids peroxidized, and membranes disrupted (24). As mitochondria are the primary source of ROS, mitochondria are also the initial targets of ROS (25, 26) that peroxidize polyunsaturated fatty acids in the mitochondrial membrane (including linoleic acid and arachidonic acid) to cytotoxic aldehydes such as MDA and other compounds (27, 28). These aldehydes are more stable than ROS; they diffuse throughout tissues to amplify the effects of oxidative damage. Reactive aldehydes change the amino acid residues of proteins, causing further damage (29). More importantly, aldehydes directly inhibit the mitochondrial respiratory chain, triggering a vicious cycle that kills many cells (30). Given the seriousness of I/R injury, drugs that reduce ROS and aldehyde production and protect mitochondrial function are urgently required.

ALDH2 (a mitochondrial aldehyde-oxidizing enzyme) detoxifies aldehydes generated by oxidative action by metabolizing them to carboxylic acids (of lower toxicity), and also aids the removal of ROS-generated aldehyde adducts (31). An increase in ALDH2 activity preserves mitochondrial function and protects against I/R injury caused by excessive ROS and aldehydes (32). The ALDH2-selective activator Alda-1 directly binds to, and activates, ALDH2; this reduced the cardiac infarct size by 60%, significantly decreased necrotic liver areas, and effectively alleviated intestinal I/R injury (8, 10, 11). We found that Alda-1 administered to rats for 7 consecutive days significantly increased the SOD level and reduced MDA production, thus reducing the accumulation of reactive aldehydes, alleviating oxidative stress, and lowering the extent of skin flap necrosis. The effects of Alda-1 on I/R injury may involve the action of the drug on PINK1/Parkin-dependent signaling. This is one of the three classical pathways of mitophagy, and is triggered by hypoxemia and ROS overproduction (33). When mitochondria become dysfunctional, PINK1 (a protein of the serine/threonine kinase family) serves as an upstream molecular sensor that accumulates on the outer mitochondrial membrane to recruit and activate Parkin (an E3 ubiquitin ligase). Parkin selectively clears damaged mitochondria by conjugating ubiquitin to mitochondrial proteins; these are then degraded in autophagosomes (34). We immunohistochemically explored the PINK1 and Parkin levels. The PINK1 and Parkin immunostaining intensities were markedly decreased by Alda-1. Thus, inhibition of PINK1/Parkin-mediated mitophagy by Alda-1 may protect skin flaps from I/R injury.

Beclin-1, a crucial regulator, plays an important role in the initiation, autophagosome fusion, and proteolytic degradation of mitophagy (35). p62, a key mediator that functions in mitophagy, can recognize ubiquitinylated outer mitochondrial membranes and promotes the formation of autophagosomes, so it is regarded as a mitophagy marker (36). Studies have shown that LC3 is an autophagosome marker that represents mitophagy activity since the amount of LC3 has significant associations with the contents of autophagic vacuoles (37). As mitophagy has been linked to the expression of Beclin-1, LC3, and p62, in our experiment, low expression levels of those markers reflect lower mitophagy activity, which corresponds to the down-regulation of the PINK1/Parkin signaling pathway.

Notably, mitophagy exhibits two (paradoxically) opposite behaviors during I/R injury. On the one hand, a certain level of mitophagy is cytoprotective; degrading mutated DNA, clearing misfolded proteins, and removing depolarized membranes, thus maintaining normal mitochondrial structure and function (38).Wang et al. (39) convincingly demonstrated that, on the activation of PINK1/Parkin-mediated mitophagy, the removal of damaged mitochondria ameliorated neuronal I/R injury in the cerebrum. However, other studies suggest that mitophagy plays a role in injury development. For example, Ji et al. (14) reported that excessive activation of PINK1/Parkin-mediated mitophagy in response to severe oxidative stress increased myocyte mortality in rats *via* myocardial I/R. Given the organ-specific differences in sensitivity and specificity, it is difficult to determine whether mitophagy activation or inhibition enhances cell survival. Additional studies are required.

I/R injury and mitophagy aside, inflammation also plays a pivotal role during flap necrosis. Inflammatory cytokines (especially IL-1β, IL-6, and TNF-α) are key mediators of inflammatory responses. IL family peptides greatly affect the initiation and maintenance of an inflammatory response. In particular, IL-1β and IL-6 induce the production of other inflammatory cytokines and inflammatory cell differentiation (40, 41), increase vascular permeability and inflammatory cell infiltration, and cause the swelling, pain, and redness of inflammatory responses (42). TNF-α was initially described as a material exerting a necrotic effect on tumor cells (hence the name) (43). TNF-α activates neutrophils and stimulates macrophages and NK cells; further, TNF-α can induce cell death characterized by cell swelling, membrane rupture, and organelle lysis (44). Diao et al. (45) found that, by activating ALDH2, Alda-1 inhibited inflammatory cytokine overproduction and hypersecretion, thus reducing cardiac and neurological injury after resuscitation. Cao et al. (46) showed that ALDH2 protected lung tissue from an excessive immune response by decreasing inflammatory cytokine release in a model of sepsis-induced pulmonary injury. We found that Alda-1 reduced neutrophil infiltration and the levels of inflammatory cytokines (IL-1β, IL-6, and TNF-α), suggesting that ALDH2 activation aided flap viability by alleviating inflammatory responses.

A blood supply with adequate oxygen and nutrients is essential for flap survival; both blood vessel “quality” and “quantity” are important. Oxidative stress is a risk factor for both vasoconstriction (47) and venous thrombosis (48). Tajima et al. (49) showed that ROS may indirectly increase platelet reactivity and contract smooth muscle cells, thus increasing the risk of blood clots. Lang et al. (50) indicated that ALDH2 may trigger vasodilation. Yasue (51) reported that individuals with a variant ALDH2 genotype (associated with a significant decrease in enzymatic activity) were more likely to experience coronary spasm and acute myocardial infarction than others, attributable to endothelial destruction, vasoconstriction, and thrombosis. We found that ALDH2 activation increased skin flap blood perfusion, as revealed by laser Doppler flowmetry; the “quality” of vessels improved. VEGF is essential to increase the “quantity” of blood vessels. VEGF promotes endothelial cell differentiation and migration; such cells form blood vessel walls (52). VEGF is thus critically important in terms of enhancing vascular angiogenesis (the process by which new blood vessels form). A previous study showed that Alda-1 increased hippocampal VEGF levels and reduced depressive-like behaviors in depressed rats after myocardial infarction (53). We found that, compared to the control group, the Alda-1 groups expressed higher levels of MVD and VEGF, and evidenced enhanced angiogenesis (as revealed by lead oxide-gelatin angiography). Thus, Alda-1 promoted skin flap microcirculation and neovascularization.

However, our work had certain limitations. Although it has been demonstrated in our experiment that the anti-oxidative action of Alda-1 facilitated flap survival and it was related to mitophagy, the complex biological process of mitophagy requires further investigation. Also, as rat and human skin differ, extensive clinical trials are required to determine appropriate Alda-1 doses, and to identify any side effects, before human testing.

## 5 Conclusion

Activation of ALDH2 significantly enhanced random skin flap survival by regulating several key processes. ALDH2 activation alleviated I/R injury, reduced inflammatory responses, promoted angiogenesis, and inhibited mitophagy. Treatment with Alda-1 may downregulate the PINK1/Parkin signaling pathway and enhance skin flap viability after flap transplantation in humans.

## Data availability statement

The original contributions presented in the study are included in the article/**Supplementary Material**. Further inquiries can be directed to the corresponding authors.

## Ethics statement

The animal study was reviewed and approved by The Laboratory Animal Ethics Committee of Wenzhou Medical University, Wenzhou, China.

## Author contributions

Conceptualization, XW; Methodology, KW; Software, JC; Data Curation, JC and HL; Investigation, YiL; Formal analysis, YiL, ZM, and QL; Validation, ZJ and XL; Writing - Original Draft, TZ; Writing - Review and Editing, YuL; Visualization, YuL and SW; Resources, DL; Supervision, DL; Project administration, DL; Funding acquisition, DL. All authors contributed to the article and approved the submitted version.
