# Supplementary material for: Activation of aldehyde dehydrogenase-2 improves ischemic random skin flap survival in rats
Source: Front Immunol. 2023 Jun 27;14:1127610. doi: 10.3389/fimmu.2023.1127610 (PMC10335790; doi:10.3389/fimmu.2023.1127610)
Supplement: Supplementary file 1 [file DataSheet_1.zip › Figure PDF/Additional Figures.pdf]

Control Group

×100 ×200

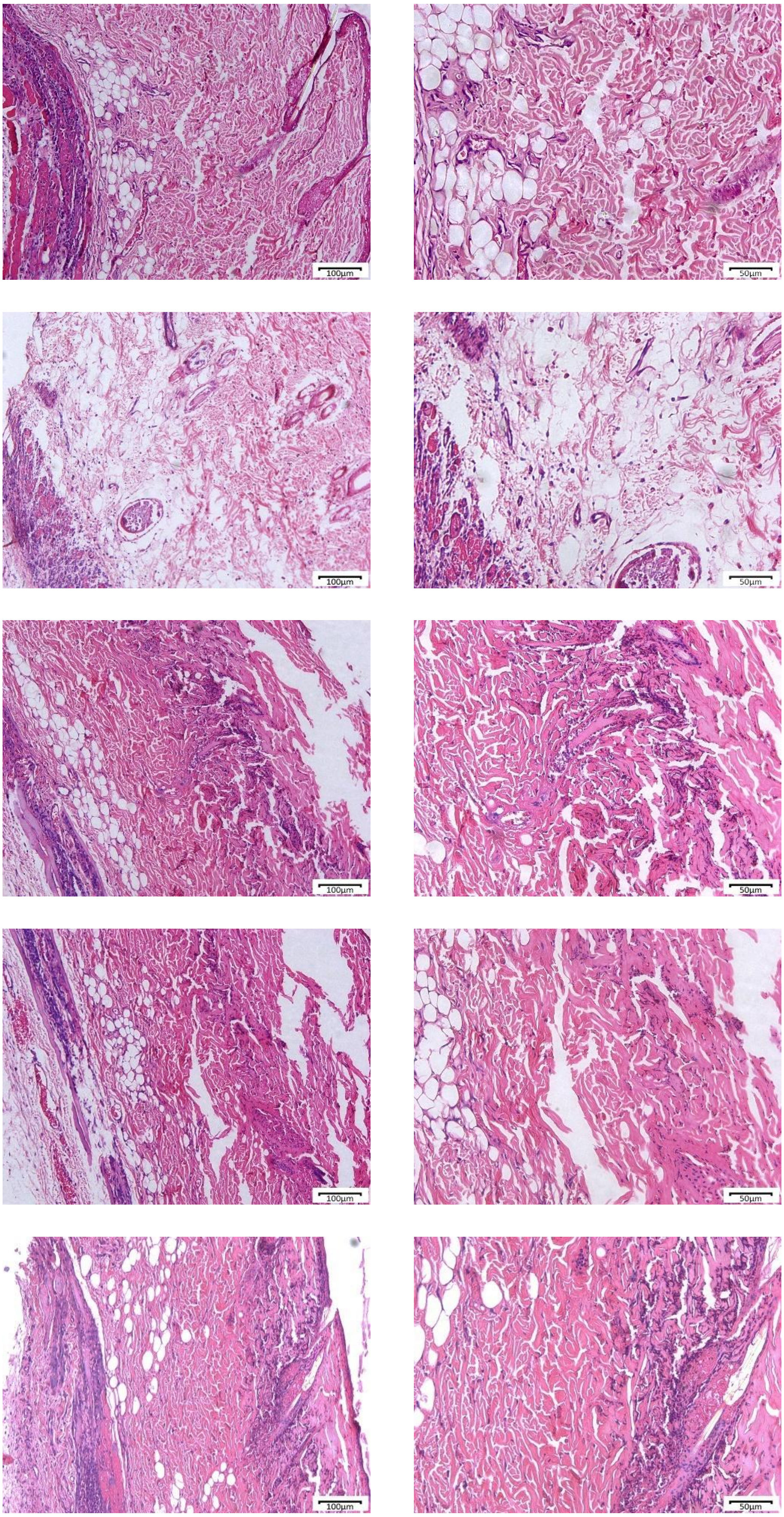

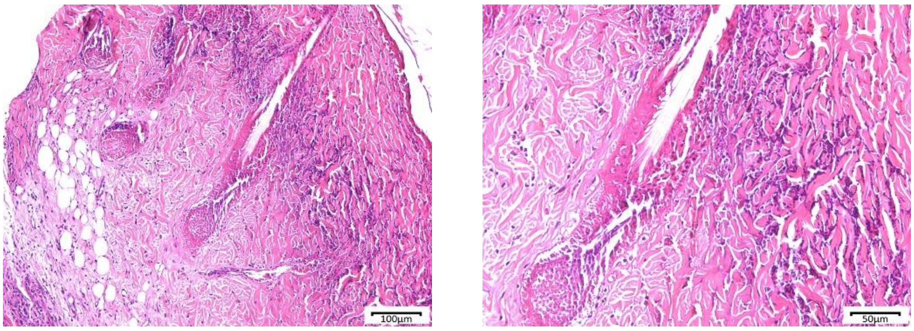

Low-dose Alda-1 Group

×100 ×200

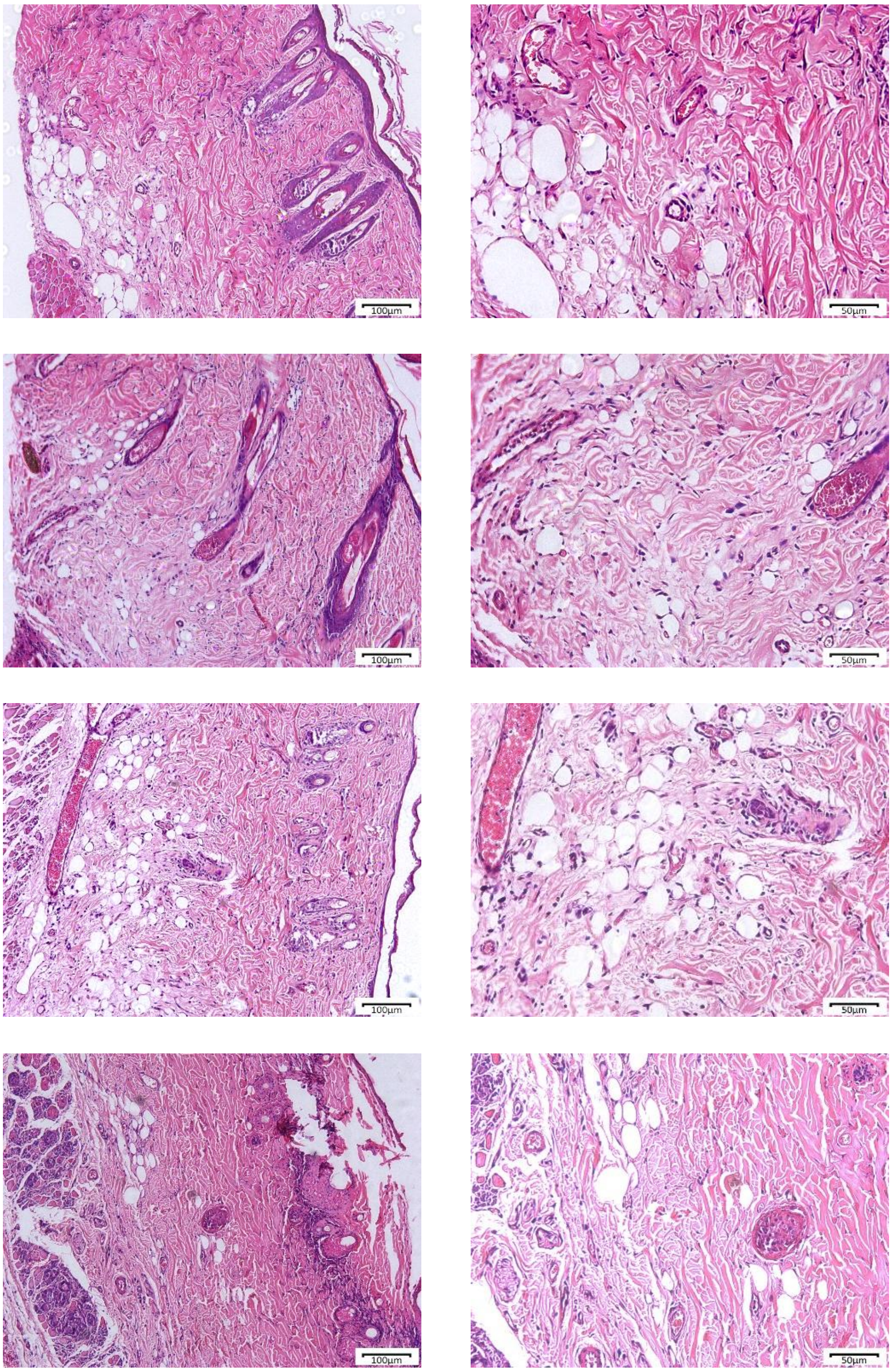

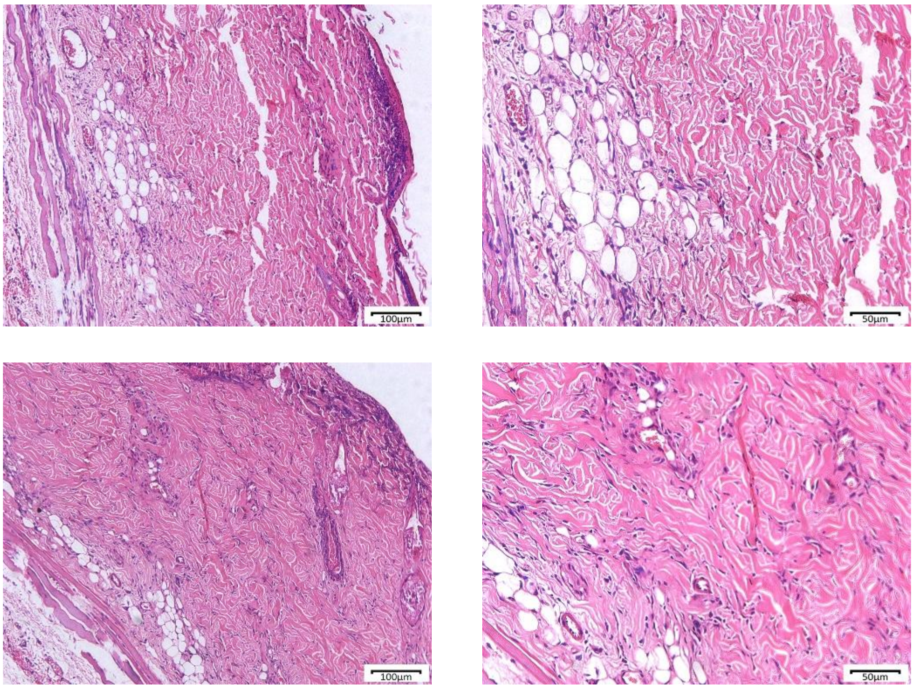

High-dose Alda-1 Group

×100 ×200

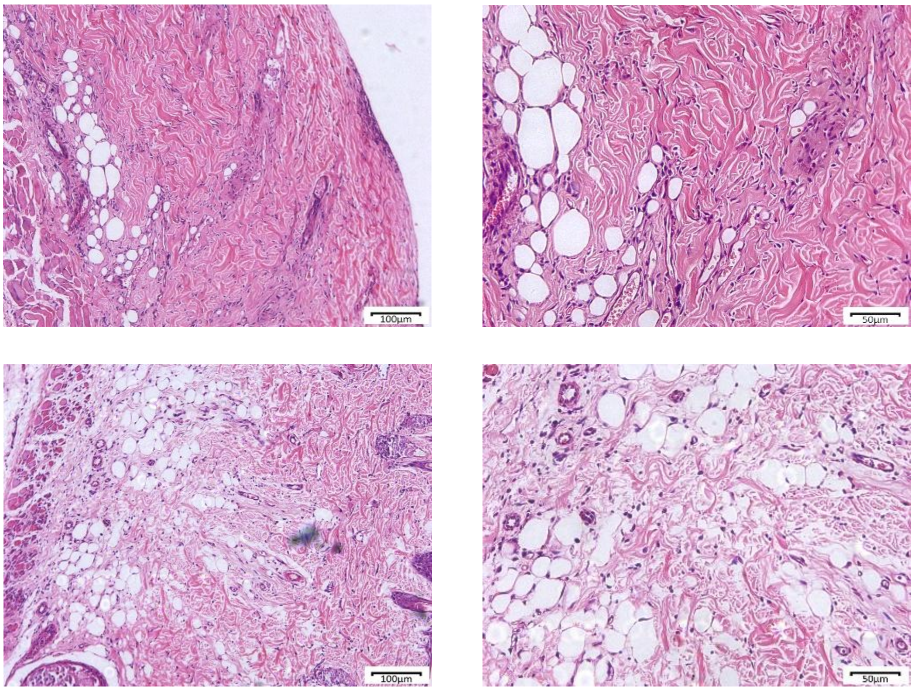

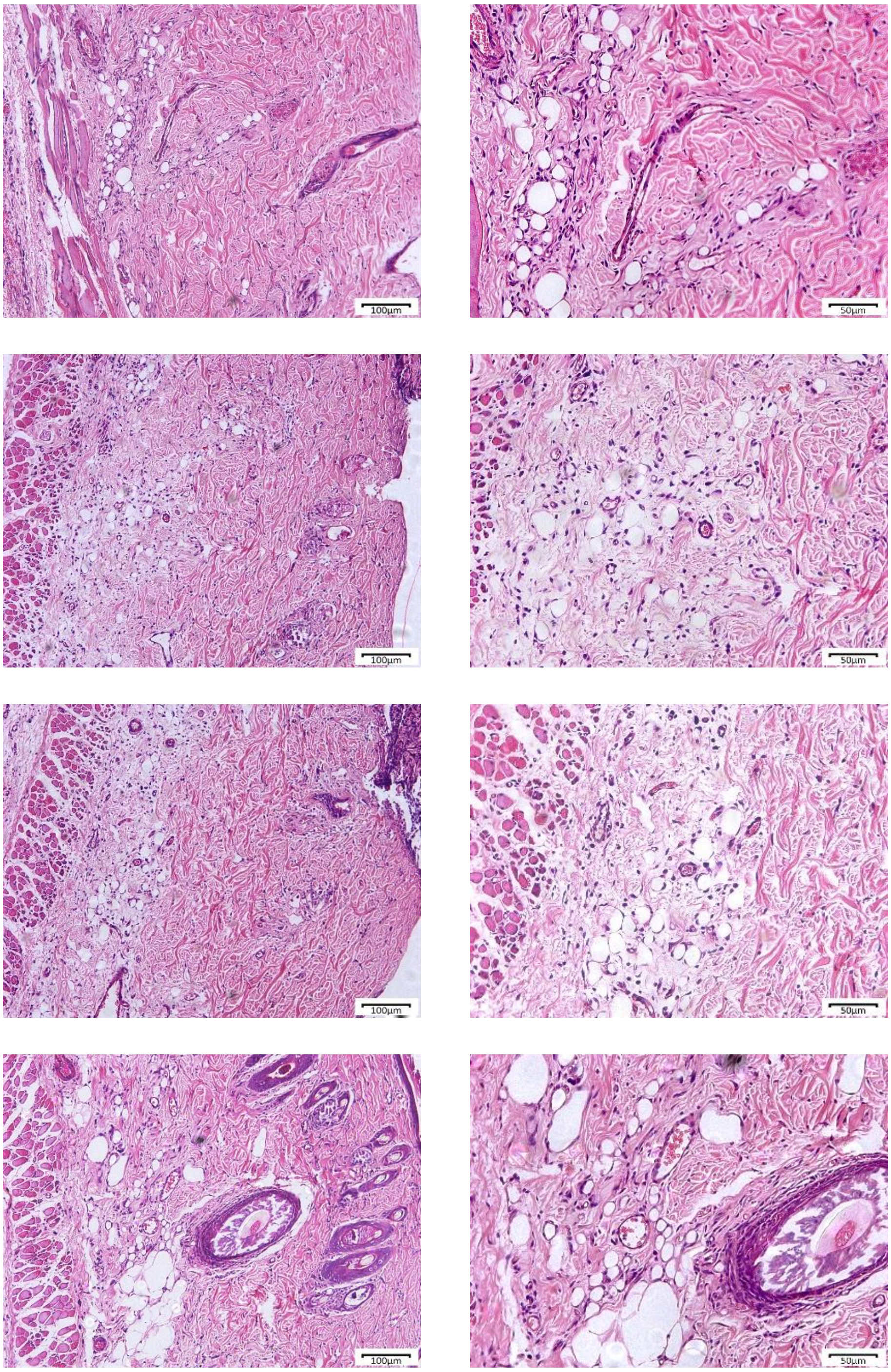
100μm
50μm
100μm
50μm
100μm
50μm
100μm
50μm

IL-1β

Control Group

Low-dose Alda-1 Group

High-dose Alda-1 Group

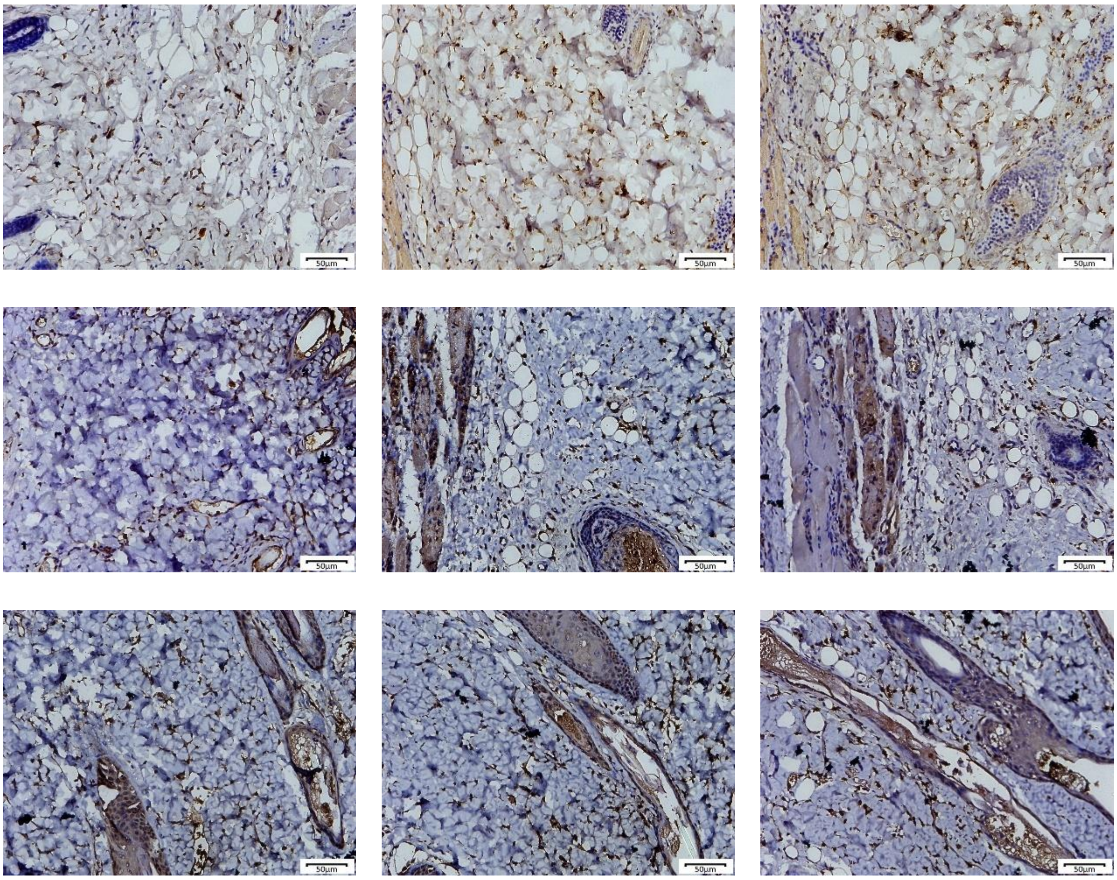

IL-6

Control Group

Low-dose Alda-1 Group

High-dose Alda-1 Group

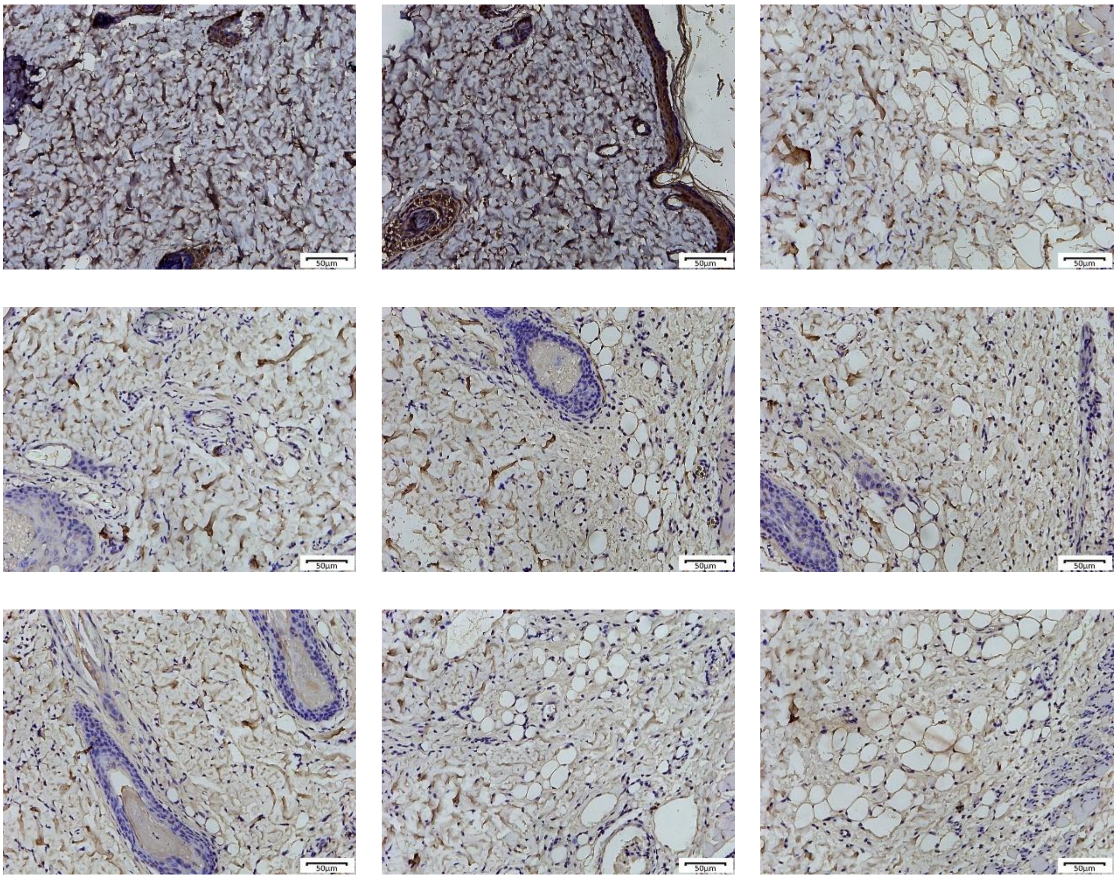

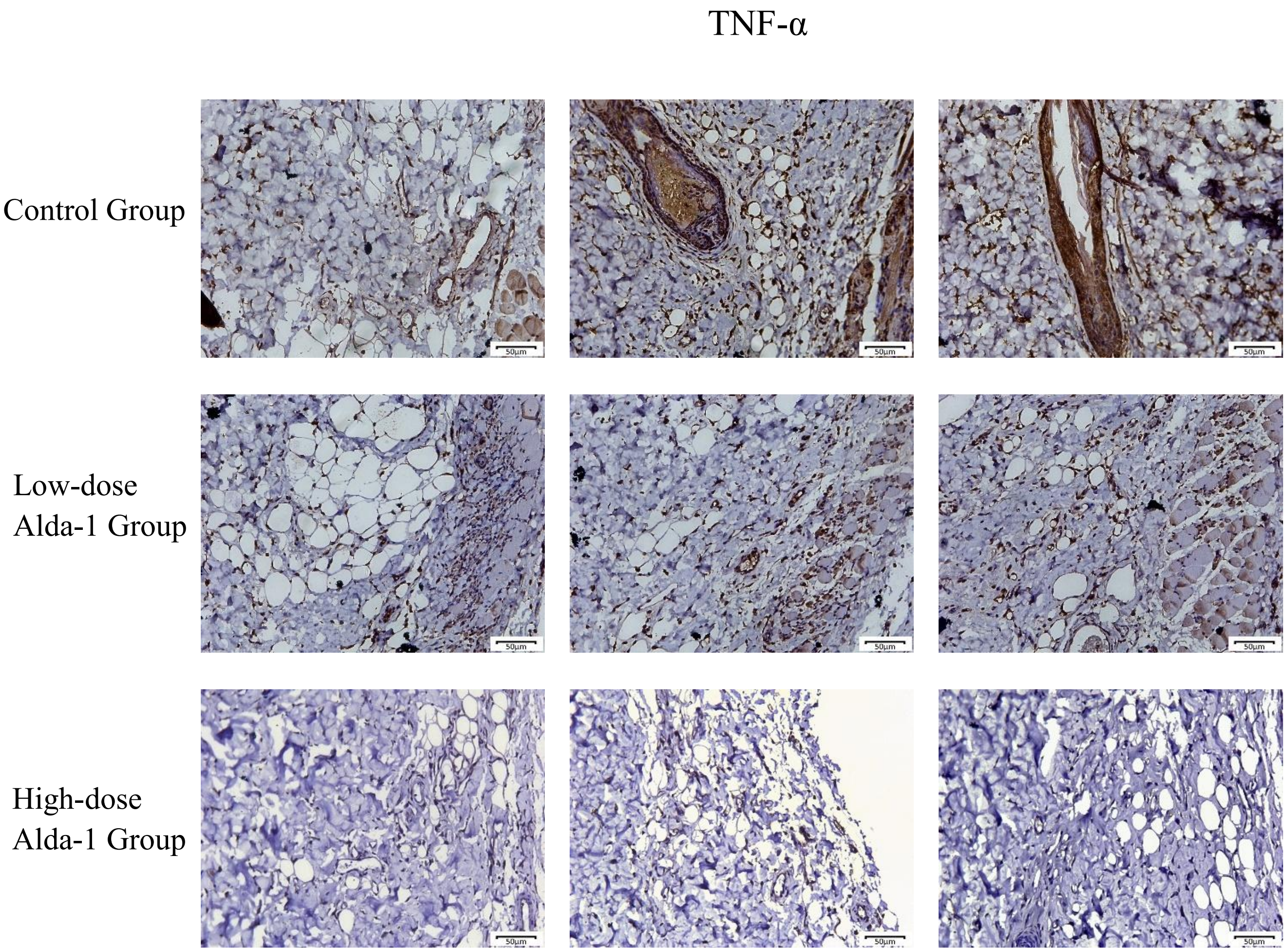
TNF-α
Control Group
Low-dose
Alda-1 Group
High-dose
Alda-1 Group

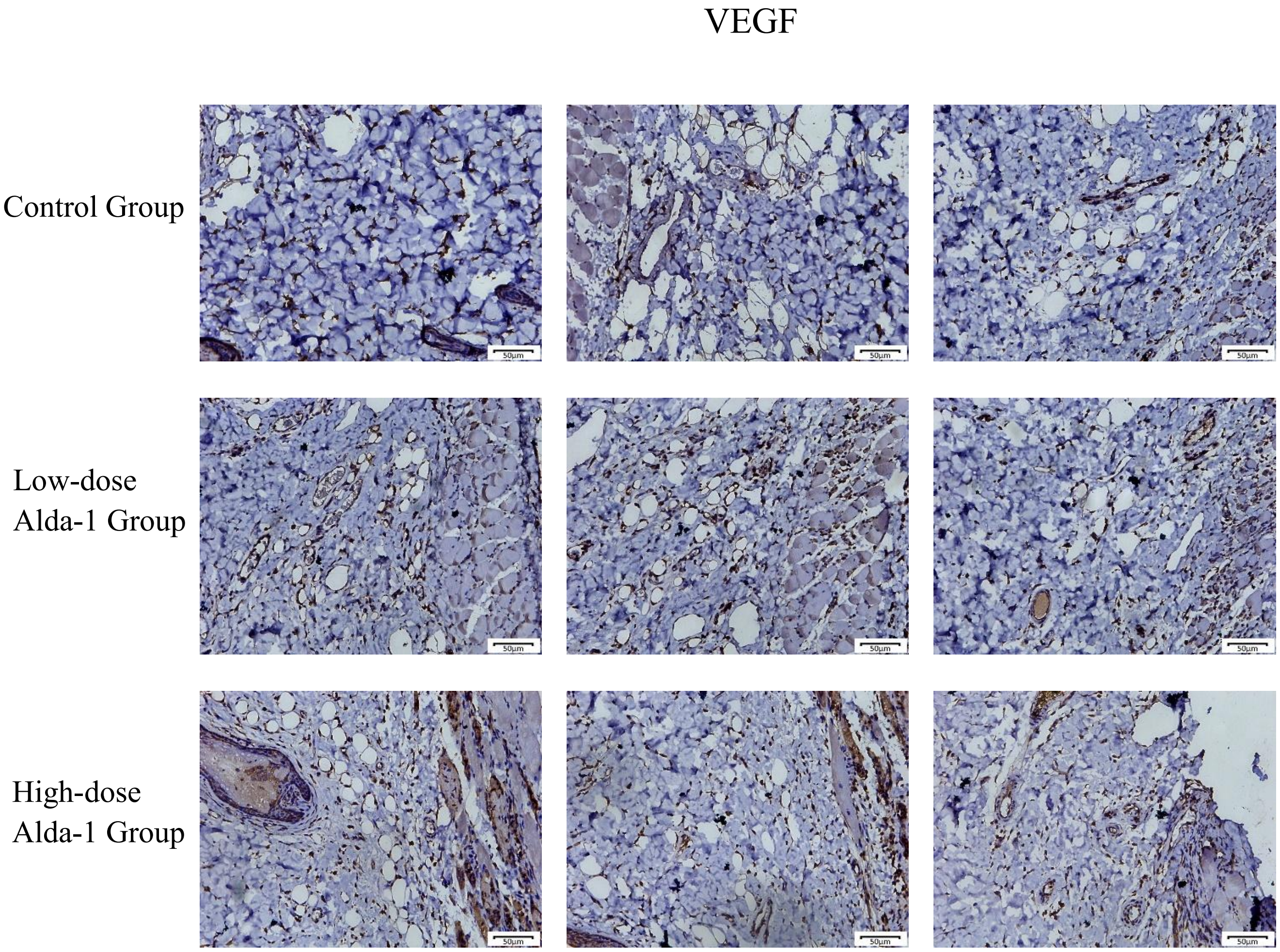
VEGF
Control Group
Low-dose Alda-1 Group
High-dose Alda-1 Group

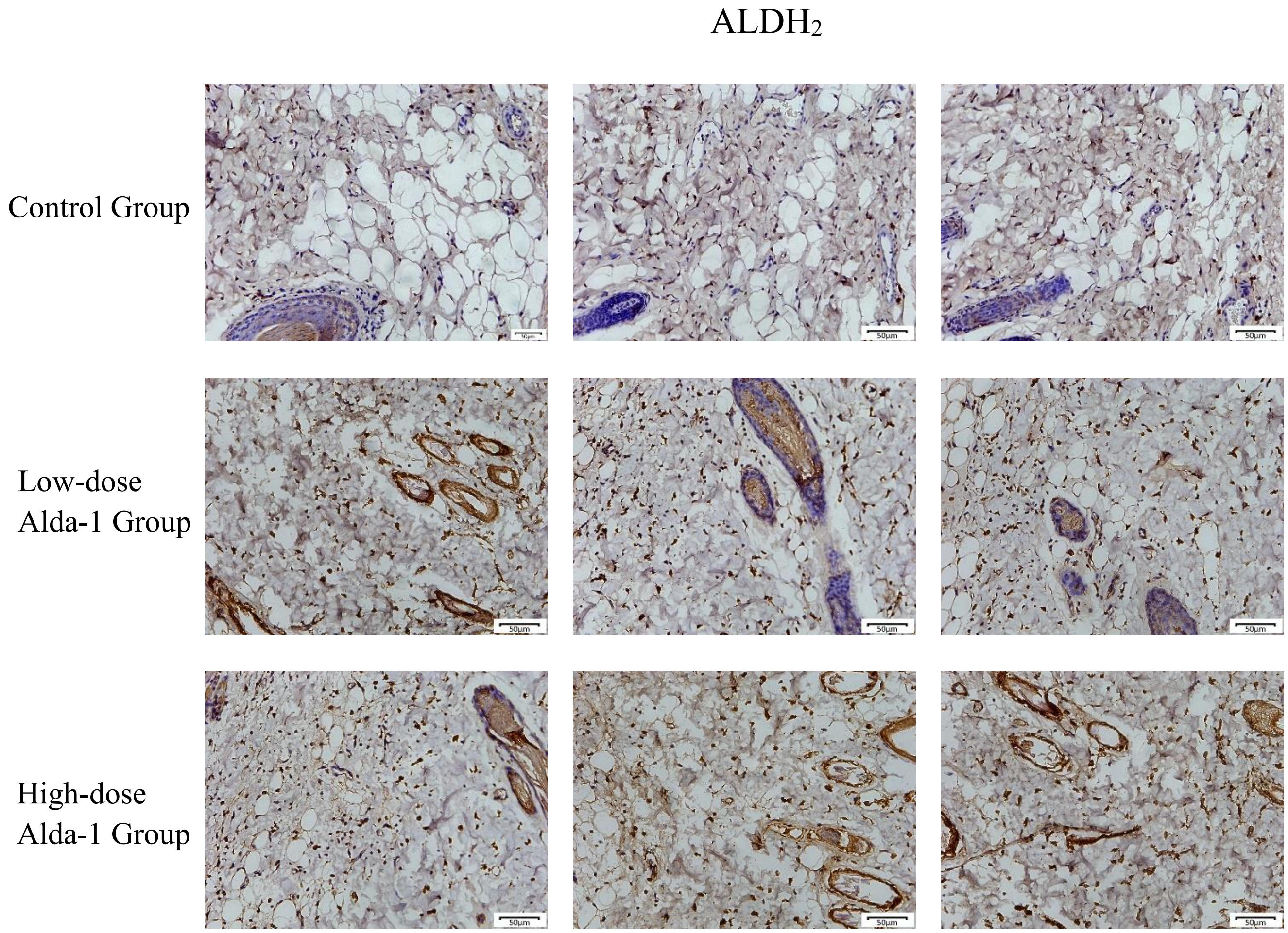
ALDH2
Control Group
Low-dose
Alda-1 Group
High-dose
Alda-1 Group
50μm

PINK1

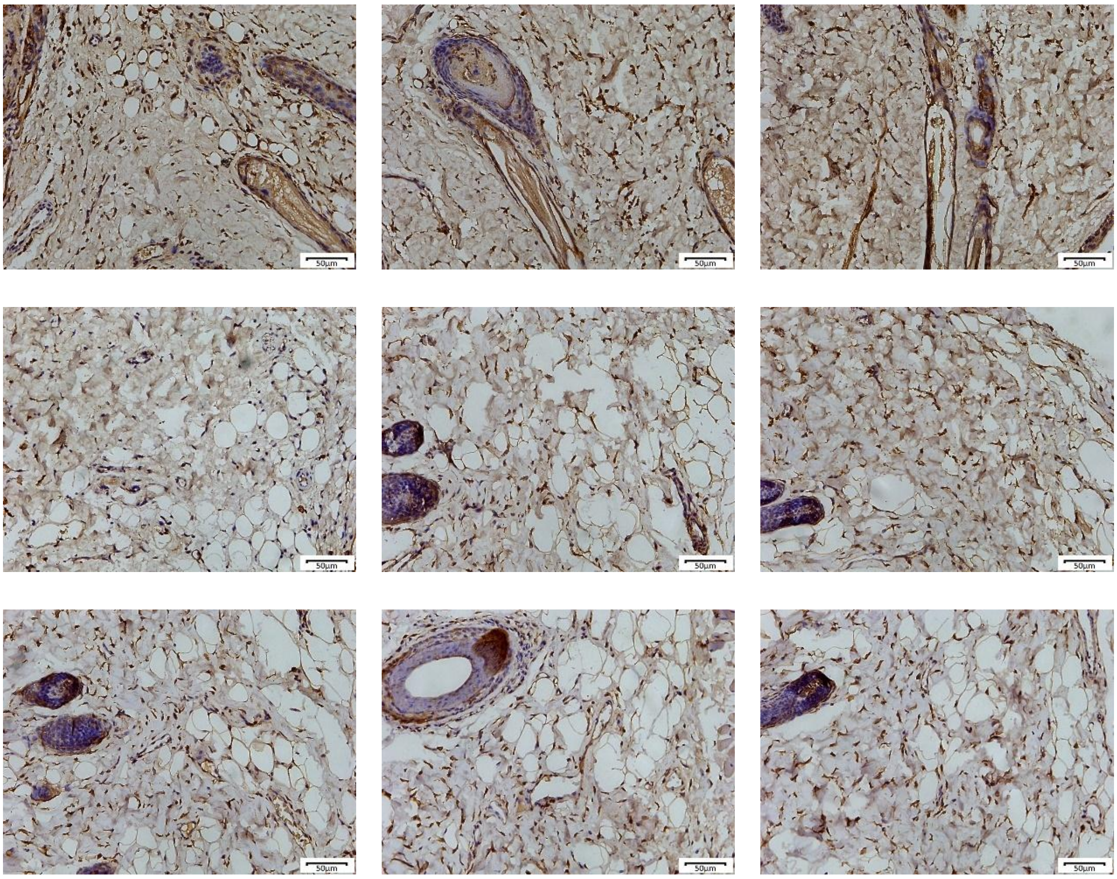

Control Group

Low-dose
Alda-1 Group

High-dose
Alda-1 Group

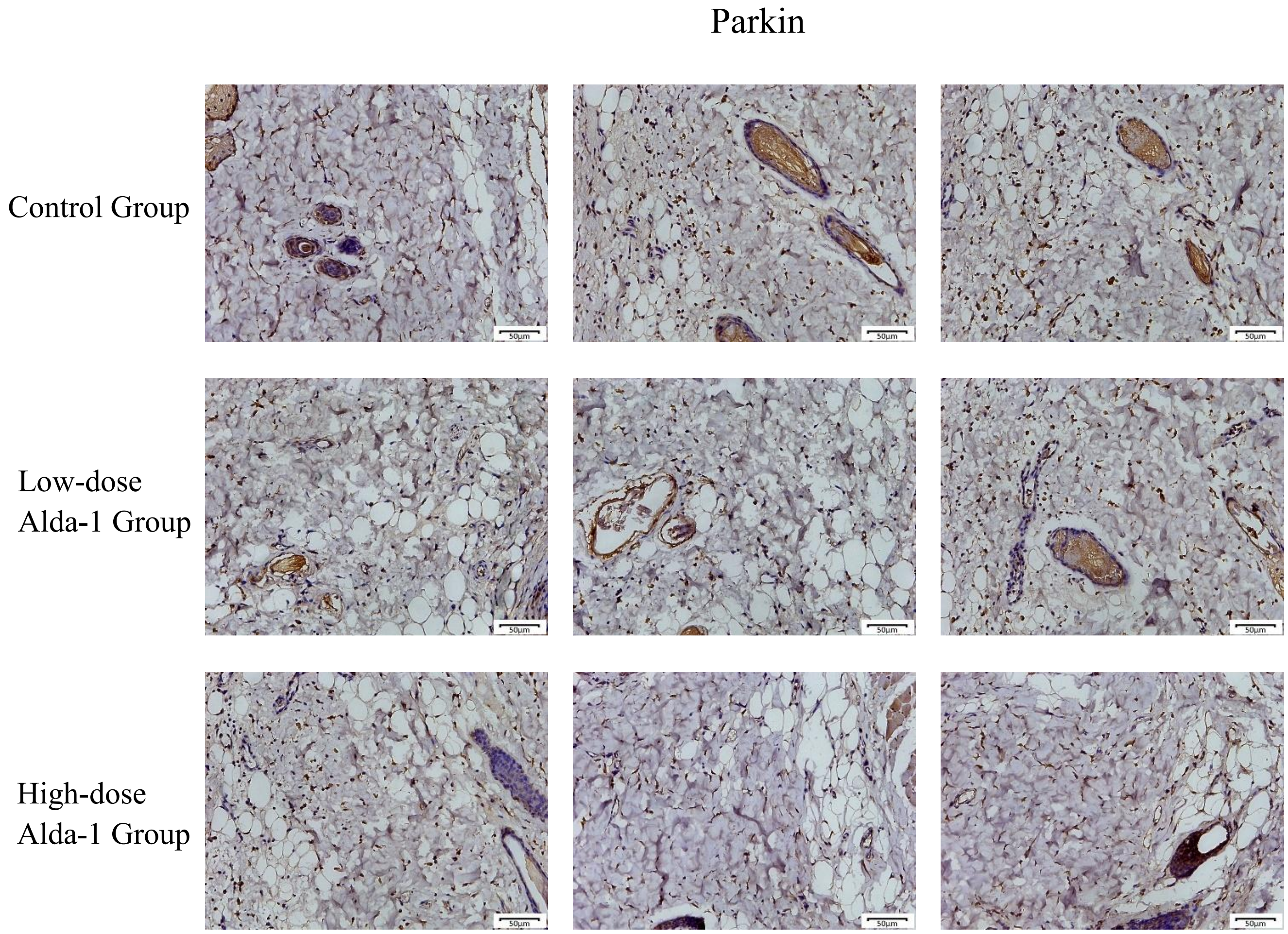
Parkin
Control Group
Low-dose Alda-1 Group
High-dose Alda-1 Group
50μm
