# Supplementary material for: Activation of aldehyde dehydrogenase-2 improves ischemic random skin flap survival in rats
Source: Front Immunol. 2023 Jun 27;14:1127610. doi: 10.3389/fimmu.2023.1127610 (PMC10335790; doi:10.3389/fimmu.2023.1127610)

Control Group

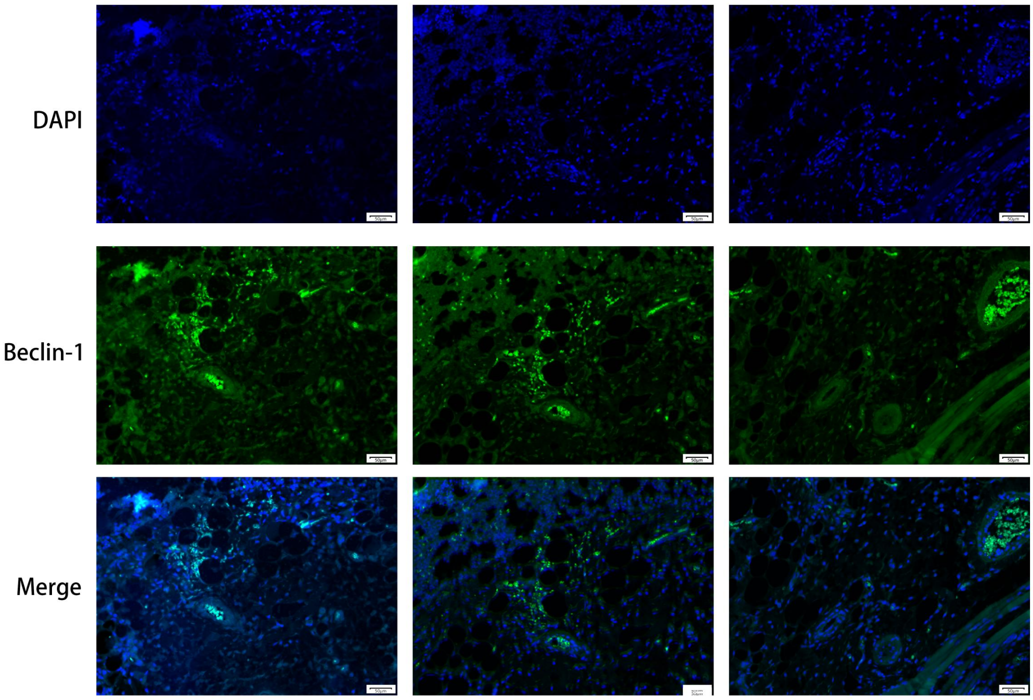

Low-dose Alda-1 Group

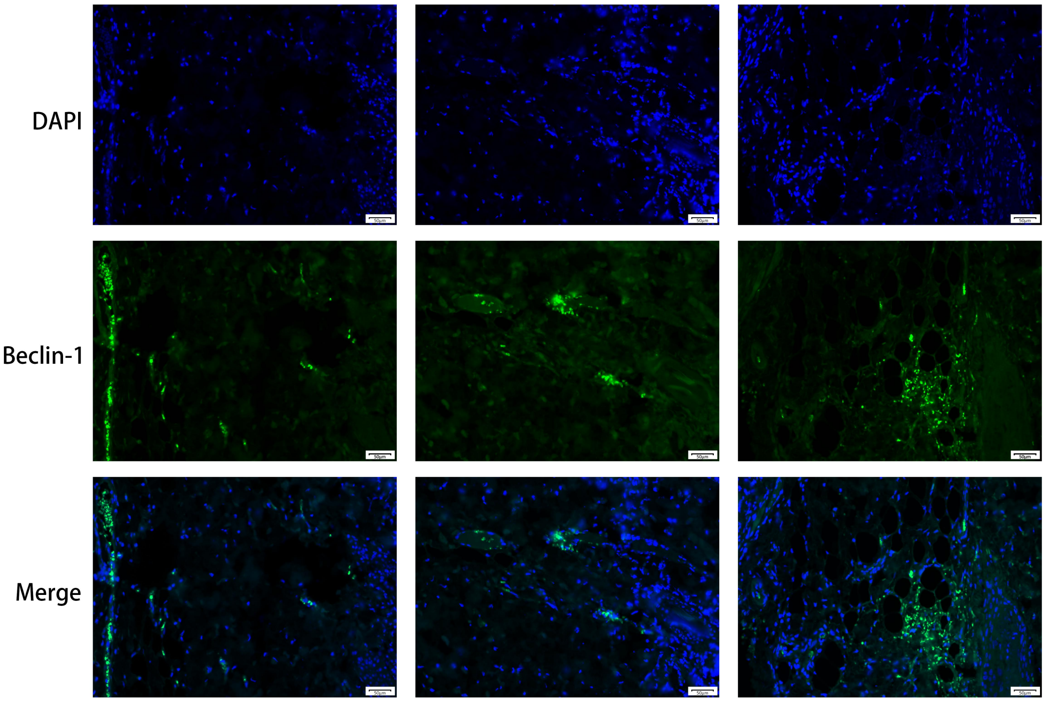

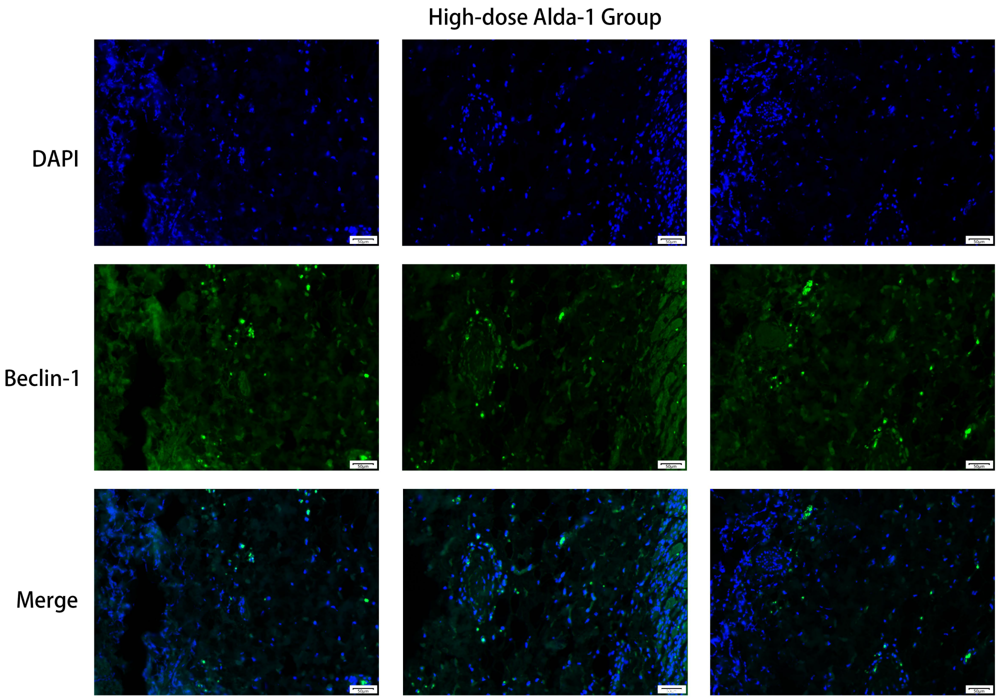
High-dose Alda-1 Group
DAPI
Beclin-1
Merge

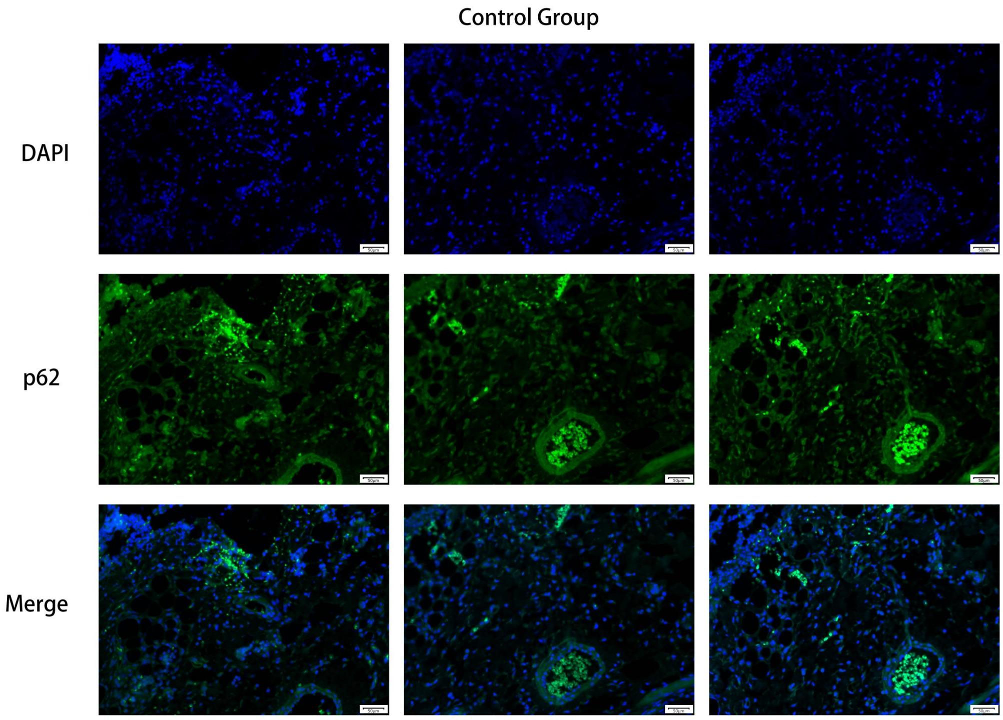
Control Group
DAPI
p62
Merge

Low-dose Alda-1 Group

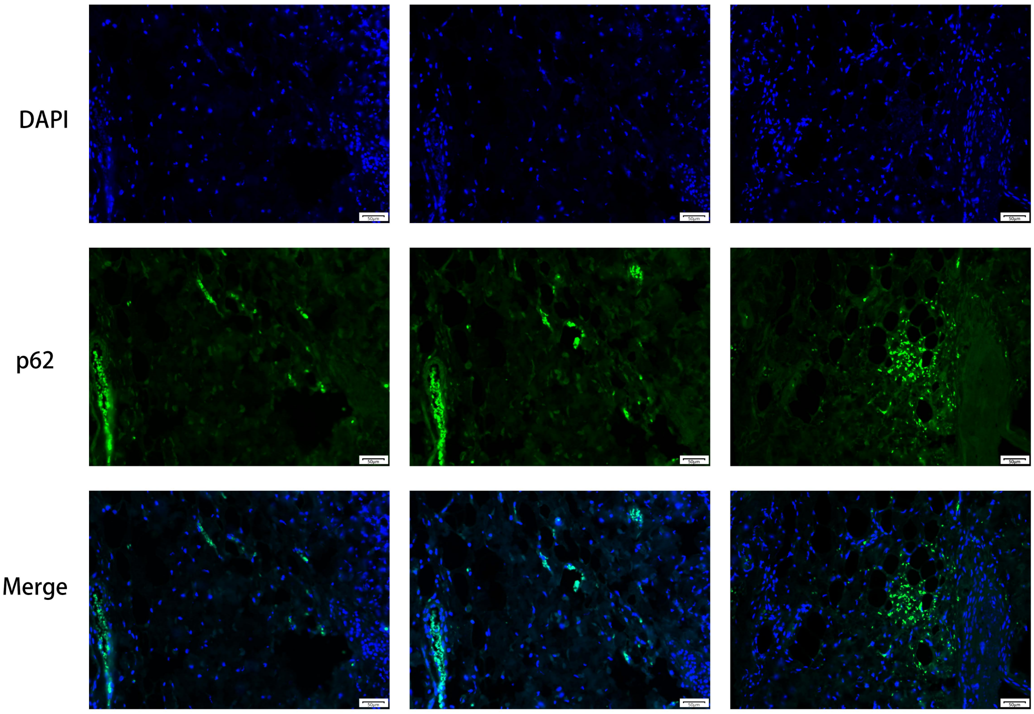

High-dose Alda-1 Group

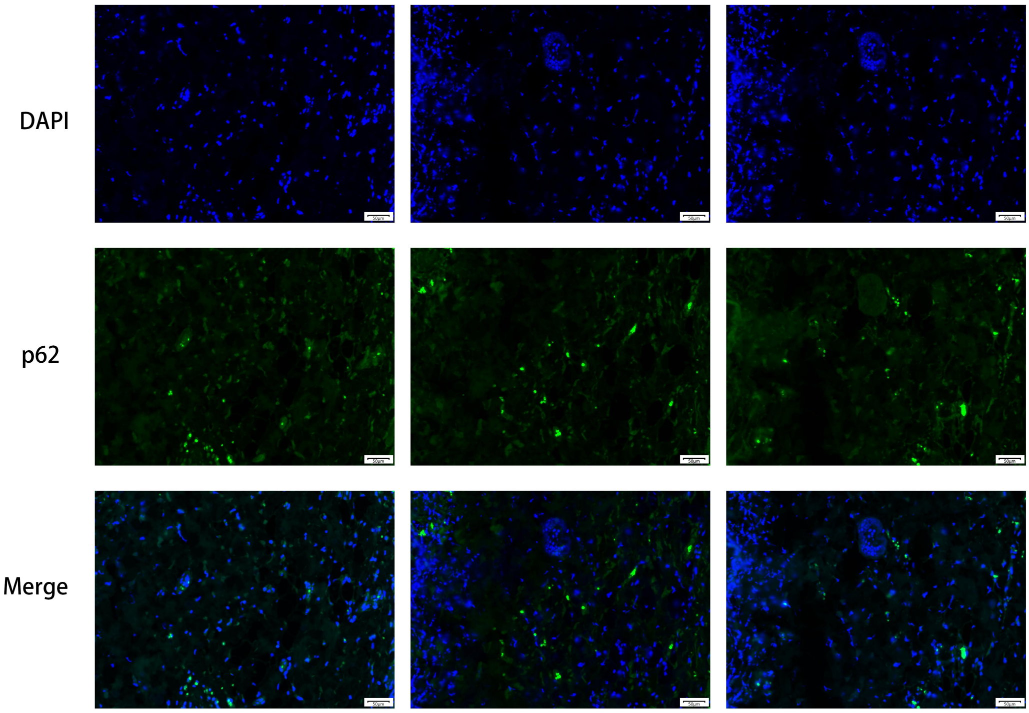

Control Group

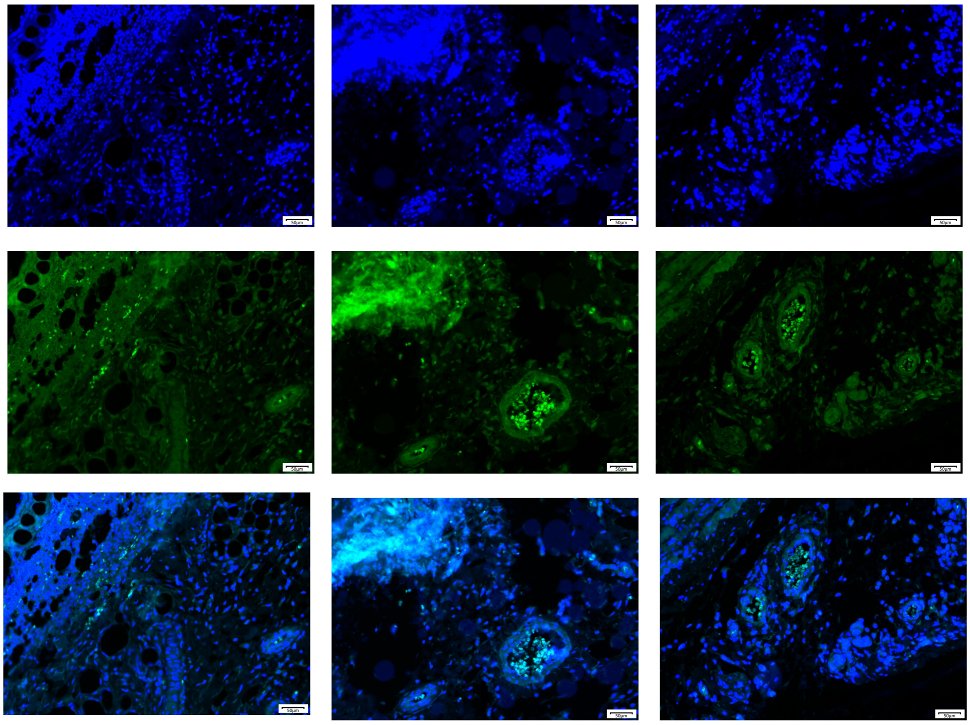

Low-dose Alda-1 Group

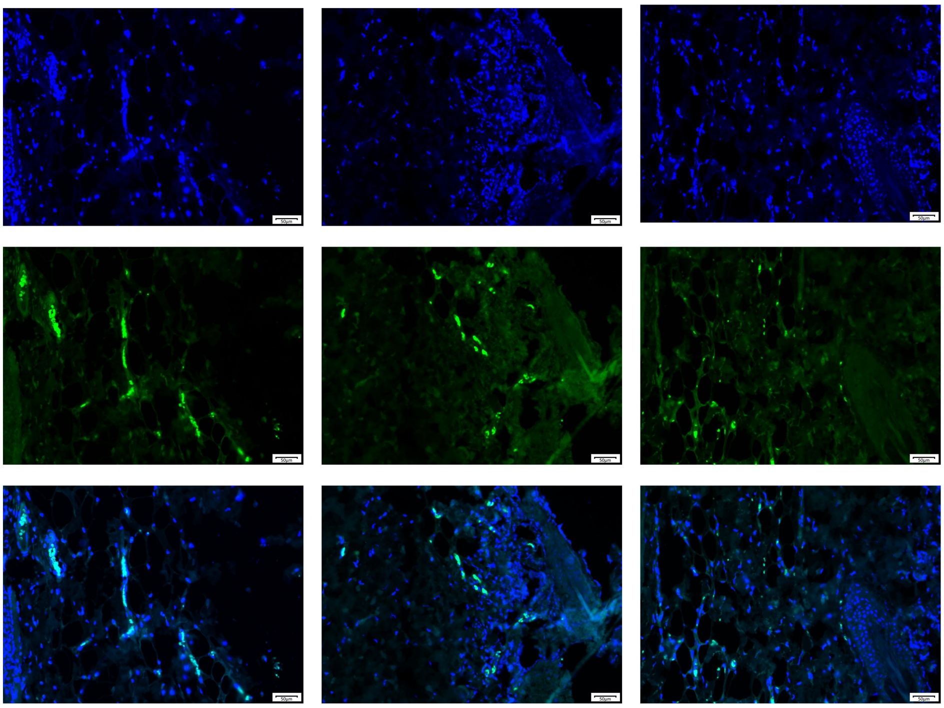

High-dose Alda-1 Group

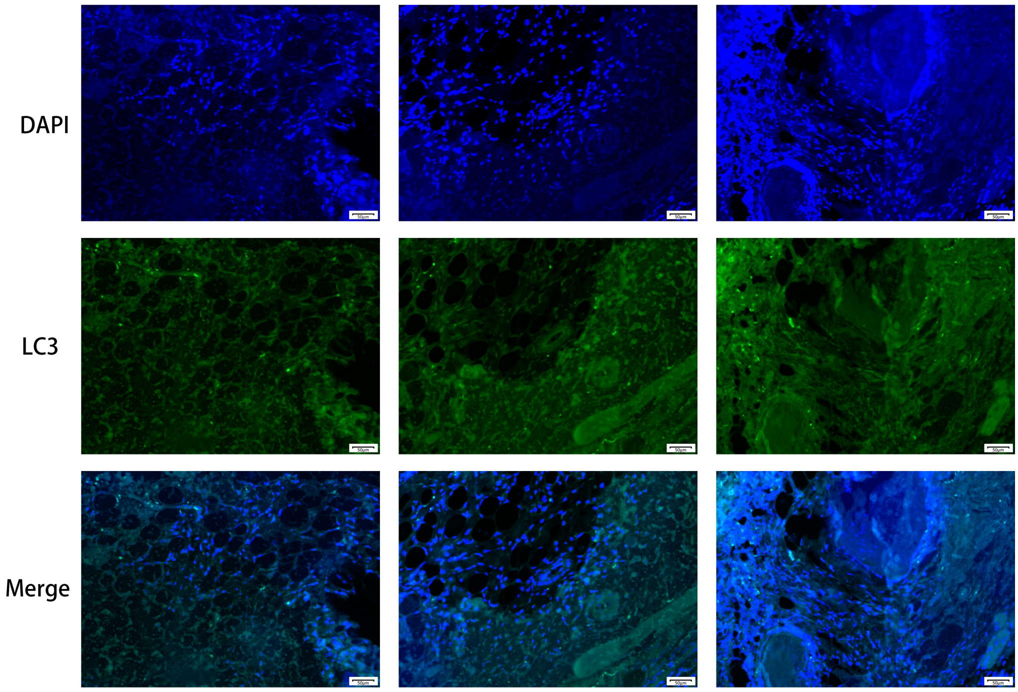

Supplement: Supplementary file 1 [file DataSheet_1.zip › Figure PDF/Additional Figures(Immunofluorescence ).pdf]
